# Individualised treatment effects of corticosteroids in IgA nephropathy

**DOI:** 10.1016/j.ebiom.2026.106396

**Published:** 2026-07-14

**Authors:** David L. Hölscher, Nikolas E.J. Schmitz, Leon Niggemeier, Pourya Pilva, Martin Strauch, Vladimir Tesar, Jonathan Barratt, Ian S.D. Roberts, Rosanna Coppo, Laura Barisoni, Motoko Yanagita, Ulas Alabalik, Andrew D. Rule, Jaidip M. Jagtap, Eliabe S. Abreu, Claudia Seikrit, Saskia von Stillfried, Maarten W. Taal, Philip A. Kalra, Juergen Floege, Rafael Kramann, Peter Boor, Roman D. Bülow, VALIGA investigators, M.L. Russo, S. Troyanov, H.T. Cook, I.S.D. Roberts, V. Tesar, D. Maixnerova, S. Lundberg, L. Gesualdo, F. Emma, F. Diomedi, G. Beltrame, C. Rollino, A. Amore, R. Camilla, L. Peruzzi, M. Praga, S. Feriozzi, R. Polci, G. Segoloni, L. Colla, A. Pani, D. Piras, A. Angioi, G. Cancarini, S. Ravera, M. Durlik, E. Moggia, J. Ballarin, S. Di Giulio, F. Pugliese, I. Serriello, Y. Caliskan, M. Sever, I. Kilicaslan, F. Locatelli, L. Del Vecchio, J.F.M. Wetzels, H. Peters, U. Berg, F. Carvalho, A.C. da Costa Ferreira, M. Maggio, A. Wiecek, M. Ots-Rosenberg, R. Magistroni, R. Topaloglu, Y. Bilginer, M. D’Amico, M. Stangou, F. Giacchino, D. Goumenos, P. Kalliakmani, M. Papasotiriou, K. Galesic, C. Geddes, K. Siamopoulos, O. Balafa, M. Galliani, P. Stratta, M. Quaglia, R. Bergia, R. Cravero, M. Salvadori, L. Cirami, B. Fellstrom, H. Kloster Smerud, F. Ferrario, T. Stellato, J. Egido, C. Martin, J. Floege, F. Eitner, A. Lupo, P. Bernich, P. Menè, M. Morosetti, C. van Kooten, T. Rabelink, M.E.J. Reinders, J.M. Boria Grinyo, S. Cusinato, L. Benozzi, S. Savoldi, C. Licata, M. Mizerska-Wasiak, G. Martina, A. Messuerotti, A. Dal Canton, C. Esposito, C. Migotto, G. Triolo, F. Mariano, C. Pozzi, R. Boero, S. Bellur, G. Mazzucco, C. Giannakakis, E. Honsova, B. Sundelin, A.M. Di Palma, E. Gutiérrez, A.M. Asunis, J. Barratt, R. Tardanico, A. Perkowska-Ptasinska, J. Arce Terroba, M. Fortunato, A. Pantzaki, Y. Ozluk, E. Steenbergen, M. Soderberg, Z. Riispere, L. Furci, D. Orhan, D. Kipgen, D. Casartelli, D. Galesic Ljubanovic, H. Gakiopoulou, E. Bertoni, P. Cannata Ortiz, H. Karkoszka, H.J. Groene, A. Stoppacciaro, I. Bajema, J. Bruijn, X. Fulladosa Oliveras, J. Maldyk, E. Ioachim, N. Bavbek, C. Alpers, F. Berthoux, S. Bonsib, V. D’Agati, G. D’Amico, S. Emancipator, F. Emmal, F. Fervenza, S. Florquin, A. Fogo, M. Haas, P. Hill, R. Hogg, S. Hsu, T. Hunley, M. Hladunewich, C. Jennette, K. Joh, B. Julian, T. Kawamura, F. Lai, C. Leung, L. Li, P. Li, Z. Liu, A. Massat, B. Mackinnon, S. Mezzano, F. Schena, Y. Tomino, P. Walker, H. Wang, J. Weening, N. Yoshikawa, C.H. Zeng, S. Shi, C. Nogi, H. Suzuki, K. Koike, K. Hirano, T. Kawamura, T. Yokoo, M. Hanai, K. Fukami, K. Takahashi, Y. Yuzawa, M. Niwa, Y. Yasuda, S. Maruyama, D. Ichikawa, T. Suzuki, S. Shirai, A. Fukuda, S. Fujimoto, H. Trimarchi, CureGN investigators, Gerald Appel, Revekka Babayev, Ibrahim Batal, Andrew Bomback, Pietro Canetta, Brenda Chan, Vivette Denise D'Agati, Samitri Dogra, Hilda Fernandez, Gabriele Gaggero, Ali Gharavi, William Hines, Krzysztof Kiryluk, Satoru Kudose, Fangming Lin, Victoria Kolupaeva, Maddalena Marasa, Glen Markowitz, Mariela Navarro-Torres, Hila Milo Rasouly, Sumit Mohan, Nicola Mongera, Jordan Nestor, Jai Radhakrishnan, Maya Rao, Maya Sabatello, Simone Sanna-Cherchi, Dominick Santoriello, Miroslav Sekulic, Michael Barry Stokes, Natalie Uy, Natalie Vena, Benjamin Wooden, Bartosz Foroncewicz, Natalia Wiewiórska-Krata, Barbara Moszczuk, Krzysztof Mucha, Agnieszka Perkowska-Ptasińska, Elżbieta Ryszkowska, Francesca Lugani, Valerio Vellone, Diego Aviles, Tarak Srivastava, Alexander Katz, Sun-Young Ahn, Prasad Devarajan, Elif Erkan, Hillarey Stone, Sherene Mason, Liliana Gomez-Mendez, Larry Greenbaum, Chia-shi Wang, Hong (Julie) Yin, Jens Goebel, Donald Weaver, Jill Krissberg, Jerome Lane, Cindy Pan, Ellen Cody, Samantha Martinek-Bundt, Dawson Carmean, Mary Dreher, Mahmoud Kallash, John Mahan, Samantha Sharpe, William Smoyer, Laura Biederman, Amira Al-Uzri, Sandra Iragorri, Myda Khalid, Craig Belsha, Elizabeth Onugha, Michael Braun, A.C. Gomez, Tetyana Vasylyeva, Daniel Feig, Melisha Hannah, Aftab Chishti, Jon Klein, Chryso Katsoufis, Wacharee Seeherunvong, Michelle Rheault, Craig Wong, Qassim Abid, John Barcia, Agnes Swiatecka-Urban, Sharon Bartosh, Brian Stotter, Joseph Gaut, Louis-Philippe Laurin, Virginie Royal, Mathieu Latour, Natlie (Natacha) Patey, Anand Achanti, Milos Budisavljevic, Vishwajeeth Pasham, Cybele Ghossein, Yonatan Peleg, Salem Almaani, Isabelle Ayoub, Samir Parikh, Brad Rovin, Anjali Satoskar, Anthony Chang, Huma Fatima, Jan Novak, Matthew Renfrow, Dana Rizk, Dhruti Chen, Vimal Derebail, Ronald Falk, Keisha Gibson, Dorey Glenn, Susan Hogan, Koyal Jain, J. Charles Jennette, Vanessa Moreno, Amy Mottl, Caroline Poulton, Monica Reynolds, Manish Kanti Saha, Nicole E. Wyatt, Agnes Fogo, Neil Sanghani, Jason Kidd, Selvaraj Muthusamy, Rebecca Scobell, Michelle Denburg, Amy Kogon, Kevin Meyers, Madhura Pradhan, Raed Bou Matar, John O'Toole, John Sedor, Christine Sethna, Suzanne Vento, Mohamed Atta, Serena Bagnasco, Alicia Neu, John Sperati, Sharon Adler, Tiane Dai, Ram Dukkipati, Frederick Kaskel, Kaye Brathwaite, Kimberly Reidy, Laura Malaga-Dieguez, Katherine Tuttle, Richard Lafayette, Kamal Fahmeedah, Elizabeth Talley, Michelle Hladunewich, Rulan Parekh, Carmen Avila-Casado, Daniel Cattran, Heather Reich, Meherzad Kutky, Yelena Drexler, Alessia Fornoni, Jeffrey Hodgin, Andrea Oliverio, Jon Hogan, Lawrence Holzman, Matthew Palmer, Gaia Coppock, Michael Mortiz, Juhi Kumar, Charles Alpers, J. Ashley Jefferson, Kamal Sambandam, Bethany Roehm, Cynthia Nast, Jean Hou, Laura Barisoni, Crystal Gadegbeku, Abigail Smith, Brenda Gillespie, Bruce Robinson, Matthias Kretzler, Zubin Modi, Laura Mariani, Lisa M. Guay-Woodford, The NURTuRE academic steering group, Maarten W. Taal, Paul Cockwell, Simon D.S. Fraser, Philip A. Kalra, Moin Saleem, David C. Wheeler, The AI4IgAN study group, Rosanna Coppo, Sean Barbour, Jonathan Barratt, Ian S.D. Roberts, Juergen Floege, Vladimir Tesar, Roman D. Bülow, David L. Hölscher, Hong Zhang, Muh Geot Wong, Laura Barisoni, Mark Haas, Motoko Yanagita, Keiichi Kaneko, Takeo Koshida, Sigrid Lundberg

**Affiliations:** a Institute of Pathology, RWTH Aachen University Hospital, Aachen, Germany; b Department of Nephrology and Immunology, RWTH Aachen University Hospital, Aachen, Germany; c Department of Nephrology, 1st Faculty of Medicine and General University Hospital, Charles University, Prague, Czech Republic; d John Walls Renal Unit, University Hospital of Leicester National Health Service Trust, Leicester, United Kingdom; e Department of Cardiovascular Sciences, University of Leicester, Leicester, United Kingdom; f Department of Cellular Pathology, Oxford University Hospitals National Health Service Foundation Trust, Oxford, United Kingdom; g Fondazione Ricerca Molinette, Torino, Italy; h Regina Margherita Children's University Hospital, Torino, Italy; i Department of Pathology, Division of AI & Computational Pathology, Duke University, Durham, USA; j Department of Medicine, Division of Nephrology, Duke University, Durham, USA; k Department of Nephrology, Graduate School of Medicine, Kyoto University, Kyoto, Japan; l Institute for the Advanced Study of Human Biology (WPI-ASHBi), Kyoto University, Kyoto, Japan; m Department of Pathology, Medical Faculty, Dicle University, Diyarbakir, Turkey; n Division of Nephrology and Hypertension, Mayo Clinic, Rochester, MN, USA; o Division of Epidemiology, Mayo Clinic, Rochester, MN, USA; p Centre for Kidney Research and Innovation, University of Nottingham, Derby, United Kingdom; q Renal Unit, University Hospitals of Derby and Burton NHS Foundation Trust, Derby, United Kingdom; r Department of Renal Medicine, Salford Royal Hospital, Northern Care Alliance NHS Foundation Trust, Salford, United Kingdom; s Department for Cardiology, RWTH Aachen University Hospital, Aachen, Germany

**Keywords:** Causal machine learning, Computational pathology, Pathomics, Personalised treatment

## Abstract

**Background:**

IgA nephropathy (IgAN) has diverse clinical presentations and responses to treatment. For systemic corticosteroids in particular, randomised controlled trials have reported conflicting effects, highlighting the need for individualised treatment strategies.

**Methods:**

In this retrospective cohort study, we derived and validated a causal machine learning (ML) framework to estimate individualised corticosteroid treatment effects in IgAN. Eight international cohorts, including the VALIGA, CureGN, and NURTuRE-CKD repositories, comprising 1022 patients, were analysed (derivation, n = 464; validation, n = 558). We integrated baseline clinical data, histopathological classification scores (MEST-C), and deep learning-based histomorphological biomarkers (pathomics) from digitised kidney biopsies. The framework estimated the effect of systemic corticosteroids on the composite endpoint of a ≥50% decline in estimated glomerular filtration rate or kidney failure within five years of biopsy.

**Findings:**

Across the overall study population, systemic corticosteroid therapy was not associated with a significant improvement in the composite outcome (*p* = 0·27). However, the causal ML framework revealed substantial treatment heterogeneity, identifying patients with high predicted benefit who achieved longer progression-free survival with corticosteroids (0·43 years, 95% CI 0·18–0·73, *p* < 0·01), while no benefit was observed in those with low predicted benefit (−0·005 years, 95% CI −0·3 to 0·22, *p* > 0·05). An individualised framework-guided treatment assignment was estimated to reduce systemic corticosteroid use by 60·7%. Pathomics facilitated the identification of interstitial inflammation and tubulitis as key features of corticosteroid response.

**Interpretation:**

This study demonstrates that a causal ML framework integrating clinical, histopathological, and pathomics predictors can individualise treatment assignments for systemic corticosteroids in IgAN. This approach provides a blueprint for precision therapy in IgAN, supporting AI-enhanced clinical decision-making in the era of emerging targeted treatments.

**Funding:**

German Research Foundation; European Research Council; German Federal Ministry of Education and Research; German Innovation Fund of the Federal Joint Committee; Clinician Scientist Program of the Faculty of Medicine RWTH Aachen University.

**Research in context**

**Evidence before this study**

We searched PubMed, Web of Science, and arXiv using the search terms “Individualized Treatment Effects OR “Causal Machine Learning” AND “Corticosteroid” AND “IgA Nephropathy” from database inception to June 11, 2026, without language restrictions. Our search yielded 299 results. Additionally, we used the bibliographies of the retrieved articles for literature review. We found several studies investigating histopathological scores or clinical parameters to assess corticosteroid responsiveness in IgA nephropathy (IgAN). However, we did not identify any study using machine learning or implementing deep learning-based image analysis to predict individualised treatment effects.

**Added value of this study**

Our study utilises causal machine learning to estimate individualised treatment effects in glomerular diseases. We incorporate a large international, retrospective cohort of patients with IgAN and available digitised kidney biopsies, allowing for the implementation of deep learning-based histomorphological biomarkers.

**Implications of all the available evidence**

Our study demonstrates the importance of individualising treatment decisions in IgAN for improving outcomes and minimising overtreatment. The proposed causal machine learning framework provides a scalable blueprint for precision therapy in glomerular diseases.

## Introduction

Immunoglobulin A nephropathy (IgAN) is the most common glomerulonephritis worldwide and a leading cause of kidney failure.^1^ It remains a complex and diverse entity with highly variable clinical presentations and progression rates,^1^ ranging from less than 10% to over 50%^2^ of patients developing kidney failure within 10–20 years of diagnosis. While optimised supportive care remains the cornerstone of IgAN management, patients at high risk of progression may benefit from systemic corticosteroids to reduce glomerular inflammation.^3^^,^^4^ Yet, evidence on corticosteroid efficacy from observational studies and randomised controlled trials is conflicting,^5^, ^6^, ^7^, ^8^ reflecting insufficient identification of patients most likely to benefit. Such predictive biomarkers are urgently needed, since side-effects of systemic corticosteroid treatment can be life-threatening.^6^^,^^9^

Clinical trials traditionally report the average effect of a treatment on the defined outcome, potentially obscuring important heterogeneity in individual responses. Although traditional statistical methods can identify subgroups of patients, these one-at-a-time analyses often cannot adequately capture multivariate interactions impacting treatment benefits. Causal machine learning (ML) provides an alternative approach by modelling counterfactual outcomes, which are potential outcomes under alternate treatment scenarios. This enables prediction of individualised treatment effects, i.e., the predicted difference in outcomes between treatment assignments for each individual patient based on their unique characteristics. However, reliable predictors of corticosteroid treatment benefit beyond proteinuria and kidney function have not been determined yet.

Nephropathology is a cornerstone in the diagnostic assessment for IgAN, with the Oxford classification (MEST-C) established as the standard histologic scoring system for patient risk prognostication.^10^ Although some components of the classification have been linked to corticosteroid treatment benefit,^8^^,^^11^ current Kidney Disease: Improving Global Outcomes (KDIGO) guidelines do not recommend using the Oxford classification to guide treatment decisions due to insufficient supporting evidence,^4^ likely due to the semi-quantitative scoring and inter-observer variability encountered in clinical practice.^12^

Digital histopathology images, so-called Whole Slide Images (WSIs), contain vast information that can be extracted using deep learning-based models.^13^ Pathomics enables automatic quantification of reproducible, explainable, and precise digital biomarkers directly from digitised kidney biopsies in a high-throughput fashion.^14^ These digital biomarkers can capture novel information that can be utilised to inform treatment decisions for individual patients in a precision medicine framework.

In this retrospective, worldwide multicentre study of 1022 patients with biopsy-proven IgAN, we developed and validated a causal ML framework that integrates clinical data, histopathological scores, and pathomics to estimate individualised treatment effects of systemic corticosteroids. Our approach addresses a critical gap: the need for more personalised treatment decisions that leverage novel data derived from kidney biopsies, in contrast to the current reliance on pooled treatment effects in IgAN. With the emergence of novel therapeutic agents^15^ and molecular targets,^16^ this framework might serve as a blueprint for advancing patient-tailored treatment strategies in IgAN.

## Methods

### Study population and cohort refinement

This retrospective analysis included 1022 patients with biopsy-proven IgA nephropathy from eight international cohorts used for multi-cohort derivation-validation. All eight cohorts included in this study were collected retrospectively, independently and autonomously for research purposes. The study cohorts include a secondary analysis of corticosteroids in the *European Validation Study of the Oxford Classification of IgA Nephropathy* (VALIGA) cohort,^17^ a European multi-centre cohort, a multi-centre repository (*Cure Glomerulonephropathy* (CureGN), North America and Europe^18^), a multi-centre national CKD cohort (*National Unified Renal Translational Research Enterprise,* NURTuRE-CKD, United Kingdom^19^), and five single-centre cohorts from Kyoto (Japan), Leicester (United Kingdom), Diyarbakir (Turkey), Rochester (United States of America), and Aachen (Germany).

All samples across cohorts were subject to the same defined inclusion criteria. We included patients ≥14 years of age with biopsy-proven IgA nephropathy (IgAN) without any reported secondary causes (e.g., liver disease) or IgA vasculitis, no kidney failure at time of biopsy, no missing estimated glomerular filtration rate (eGFR) at time of biopsy and at least one month of clinical follow-up.

To maintain consistency across cohorts and minimise treatment-related confounding, patients who were enrolled in the respective study over six months after kidney biopsy, received documented corticosteroid treatment prior to kidney biopsy or only after one year of follow-up, did receive other immunosuppressive agents (e.g., cyclophosphamide), targeted-release corticosteroid formulations (e.g., Nefecon) or dual endothelin and angiotensin II receptor antagonists (DERAs, e.g., Sparsentan) were excluded. Cases without sufficient number of glomeruli according to the Oxford classification for IgA nephropathy^20^ or with broken/damaged glass slides were also excluded. As multiple studies have shown a considerably increased rate in serious adverse events (SAEs) in patients with decreased kidney function ≤30 ml/min/1·73 m^21^^,^^22^ these patients were also excluded.

The VALIGA study^17^ is a multi-centre cohort across 55 participating centres in Europe including a secondary analysis of corticosteroid therapy.^8^ In total, 507 patients met the above-mentioned criteria and were considered for further analysis. The Kyoto cohort (n = 92) is a single-centre cohort from the Kyoto University Hospital in Japan with kidney biopsies taken between 2013 and 2020. The National Unified Renal Translational Research Enterprise chronic kidney disease (NURTuRE-CKD) cohort^19^ (n = 20) is a national multi-centric prospective cohort of 2996 participants from 16 participating hospitals in the United Kingdom, biopsy samples were collected between 2017 and 2019.

The Leicester cohort (n = 93) is a single-centre cohort from the University Hospital of Leicester National Health Service Trust in the United Kingdom with kidney biopsies performed from 2010 to 2023. The Aachen cohort (n = 27) is a single-centre cohort from the University Hospital RWTH Aachen in Germany with kidney biopsies from 2018 to 2025. The Diyarbakir cohort (n = 106) is a single-centre cohort from the Dicle University Hospital in Turkey. Kidney biopsies were collected from 2015 to 2024. The Rochester cohort (n = 42) is a single-centre cohort of local patients at the Mayo Clinic in Rochester (United States of America) with kidney biopsies performed between 1996 and 2015.^23^ The Cure Glomerulonephropathy (CureGN) cohort (n = 135) is a multi-centric observational study of glomerular disease including IgAN.^18^ Patients were recruited from 57 study sites in the United States of America, Canada, Italy and Poland.

**Fig. 1:**
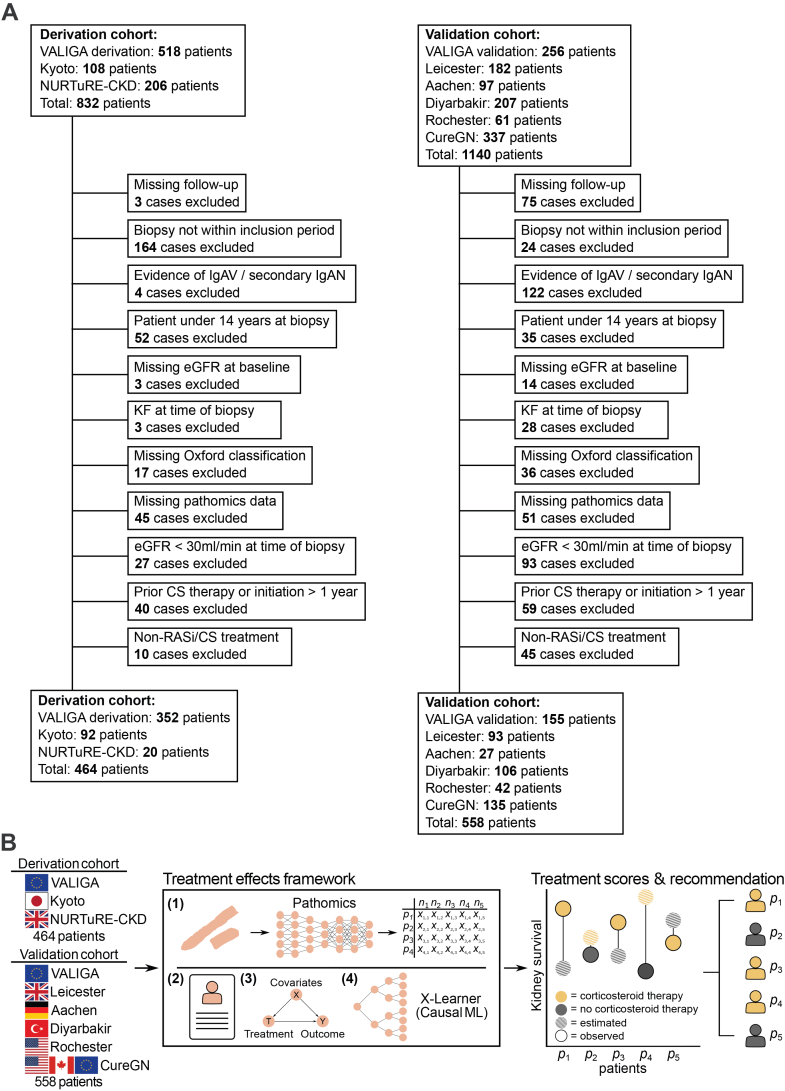
**Overview of the proposed framework for individualised treatment effects prediction.** Eight cohorts from Europe, North America and Asia were used for derivation and validation with the cohort refinement flowchart shown in **(A)**. **(B)** All patients from the eight cohorts were analysed using the causal ML framework, including (1) pathomics-derived predictors obtained via digital image analysis and (2) baseline clinical and histopathological descriptors. (3) Treatment heterogeneity based on included covariates was analysed using an X-learner algorithm (4) which is a meta-learning approach to estimate counterfactual (unobserved) outcomes for each patient under both treatment and control scenarios. Patient-level predictions of the individualised treatment effect are computed, informing the development of a personalised treatment recommendation. Abbreviations: VALIGA, European Validation Study of the Oxford Classification of IgA Nephropathy; NURTuRE-CKD, National Unified Renal Translational Research Enterprise-Chronic Kidney Disease; CureGN, Cure Glomerulonephropathy; IgAV, IgA vasculitis; IgAN, IgA nephropathy; eGFR, estimated glomerular filtration rate; KF, kidney failure; CS, corticosteroid; RASi, renin-angiotensin-system inhibitor; ML, machine learning.

In total, 1022 eligible patients were identified and divided into derivation and validation cohorts. The cohort refinement process is outlined in Fig. 1A. The VALIGA cohort was split by clinical centre into derivation and validation subsets to better balance in covariates, treatment regimens and outcomes.^24^ In summary, 464 patients from the VALIGA, Kyoto and NURTuRE-CKD cohorts formed the derivation cohort while 558 patients from the VALIGA, Leicester, Aachen, Diyarbakir, Rochester and CureGN cohorts formed the validation cohort.

### Study design

This study applied an effect-based analysis of heterogeneity of treatment effect (HTE), as outlined in the effect-modelling framework of the *Predictive Approaches to Treatment Effect Heterogeneity* (PATH) statement.^25^ Reporting of study populations, outcomes, predictors, model development, and validation adhered to the *Transparent Reporting of a Multivariable Prediction Model for Individual Prognosis or Diagnosis* (TRIPOD+AI) guideline^26^ and the *Strengthening the Reporting of Observational Studies in Epidemiology* (STROBE) statement. All patients were assessed at time of kidney biopsy with subsequent follow-up for a period of five years. Treatment was defined as initiation of systemic corticosteroid therapy within the first year after biopsy. Patients receiving other immunosuppressants, targeted-release corticosteroid formulations or dual endothelin and angiotensin II receptor antagonists (DERAs) were excluded. The primary composite outcome was progression of IgAN, defined as a sustained ≥50% decline in eGFR and/or development of kidney failure (persistent eGFR below 15 ml/min/1·73 m^2^, initiation of dialysis, or kidney transplantation) within five years of follow-up after time of kidney biopsy as this corresponds to the median follow-up duration for both derivation and validation cohorts.^27^ Outcomes were analysed using pseudo-values for the restricted mean survival time (RMST)^28^ after kidney biopsy. This approach allows for a more flexible and robust effect modelling for right-censored survival analysis.^29^

### Predictors

Demographic and clinical parameters including age, sex, race and ethnicity, body-mass-index (BMI), eGFR, proteinuria, systolic, diastolic and mean arterial blood pressure (MAP) were recorded at time of biopsy. Proteinuria, mean arterial blood pressure (MAP) and eGFR at kidney biopsy were reported as the closest values to the time of biopsy within a 180-day period. For adults, eGFR was calculated from serum creatinine using the 2021 CKD-EPI equation,^21^ while for children (<18 years of age), the Schwartz formula^30^ was applied (as done previously in VALIGA^8^). Proteinuria was reported as grams per day and taken from 24-h urine collections. Whenever these were not available, the daily urinary protein excretion was estimated from spot urine protein or albumin to creatinine ratios by using previously validated regression equations.^31^^,^^32^ Reported age corresponds to age at time of biopsy. Sex was self-reported at baseline. Race/ethnicity were collected based on reports from individual databases in accordance with local guidelines (e.g., National Health Service Ethnicity Codes^33^) and summarised into the categories ‘White’, ‘Black’, ‘South Asian’, ‘Chinese’, ‘Japanese’ and ‘Other’ (e.g., Pacific Islanders, Hispanic).^34^ Although these categories might not fully represent the granularities between patients and cannot account for social or other demographic factors, they were designed to capture potential treatment effect modifiers, as different progression rates and responses to immunosuppressive treatments have been reported in these populations.^35^

All included biopsy cases were scored locally in accordance with the Oxford classification for IgAN,^20^^,^^36^ missing cases were rescored centrally blinded to clinical and other pathological data. The MEST-C score components were defined as follows: M0/M1 as mesangial hypercellularity (≥4 mesangial cells per mesangial area) with < or ≥ 50% of glomeruli affected, E1/E0 as the presence or absence of endocapillary hypercellularity in any glomerulus, S1/S0 as the presence or absence of segmental sclerosis and/or tuft adhesions in any glomerulus and T0/T1/T2 as the degree of tubular atrophy and/or interstitial fibrosis of the cortical area (<25%, 25–50%, >50%). Crescents were only considered as the absence or presence of cellular or fibrocellular crescents (C0/C1) due to the low incidence of ≥25% glomeruli with crescents (C2) in the derivation cohort.

For all patients’ information on corticosteroid and renin-angiotensin system blocker (RASi) treatment during follow-up was collected, although reporting of dosage and formulations was inconsistent.

Missingness in baseline predictors was limited and observed only for proteinuria and MAP (Supplementary Table S1). To address this, we performed multivariate imputation via chained equations (MICE)^37^ using predictive mean matching for continuous variables with 20 multiple imputations and 10 iterations.

Additionally, we analysed five biopsy-level pathomics predictors of glomerular (glomerular tuft area, tuft circularity and tuft eccentricity) and tubulointerstitial morphometry (tubular diameter and tubular distance). These pathomics predictors were previously demonstrated to be associated with long-term outcomes in IgAN.^38^ Kidney biopsies from all included patients were digitised and analysed using our previously published framework for pathomics in Nephropathology.^38^ For better segmentation accuracy across all cohorts, we refined our previous approach to using a MedNeXt-based architecture^39^ with subsequent computation of quantitative morphometric features on instance-level. All features were then aggregated at biopsy-level using the median of the distribution.^40^ Definitions for the five pathomics predictors are provided in Supplementary Table S2. Pathomics features were not scaled or normalised between cohorts to preserve interpretability.

### X-learner framework

We applied an Xboost model to estimate heterogeneous treatment effects due to its advantages for unbalanced treatment assignment and suitability for retrospective observational studies.^41^ Xboost is an XGBoost-based implementation of the X-learner, a meta-learning framework for causal inference.^42^

The X-learner proceeds in three main steps. First, two outcome models are trained separately: one for the untreated group to estimate

$$\mu_{0}\left(X\right)=E\left[Y|T=0,X\right]\text{,}$$

and one for the treated group to estimate

$$\mu_{1}\left(X\right)=E\left[Y|T=1,X\right]\text{,}$$

where X denotes covariates, Y the outcome and T the treatment. These models predict potential outcomes for all individuals under both treatment conditions. In the second step, individual-level pseudo-treatment effects are imputed: for treated individuals, the observed outcome is compared to their predicted control outcome

$$\tau_{1}\left(X\right)=Y\left(1\right)-\mu_{0}\left(X\right)\text{,}$$

and for controls, the predicted treated outcome is compared to the observed control outcome

$$\tau_{0}\left(X\right)=\mu_{1}\left(X\right)-Y\left(0\right)\text{.}$$

These differences serve as pseudo-outcomes that are then modelled separately in each group as functions of patient covariates. In the final step, the two resulting treatment effect models are combined to form a final estimate of τ(X), using weights derived from a propensity score model that reflects the probability of being treated given covariates. This weighted averaging enhances stability, particularly in settings where the proportion of treated and untreated individuals is imbalanced.

In our implementation, gradient-boosted trees (XGBoost) were used as base learners for both outcome and treatment effect modelling due to their robustness to nonlinearities and high-dimensional data. The framework was implemented using the *rlearner* package (v1.1.0) in R.^42^

We tuned hyperparameters for all XGBoost base learners using a k-fold cross-validated randomised search with early stopping, as implemented in *cvboost*. For each X-learner submodel, the outcome regressions μ1(X) and μ0(X), the two pseudo-outcome regressions used in the X-learner step, and the propensity model p(X), were tuned separately with 10-fold cross-validation. For each submodel, we evaluated 10 randomly sampled hyperparameter configurations. Within each candidate, XGBoost was trained for up to 1000 boosting rounds with early stopping after 10 rounds without improvement on the cross-validated validation loss. Regression submodels used the root-mean-squared error (RMSE) as the evaluation metric, the propensity model was evaluated using log-loss (cross-entropy). The best configuration per submodel was selected by the lowest mean test loss across folds, and the final model was refit on the full training data using that configuration and the cross-validated optimal number of trees. The final fitted model was subsequently applied to the validation cohort.

### Model assessment

To assess whether the X-learner reliably captured treatment heterogeneity, we evaluated multiple performance metrics: the Qini coefficient, the rank-weighted average treatment effect (RATE) and the concordance statistic for benefit (C-for-benefit). For each metric, 95% confidence intervals were estimated via bootstrap resampling. To mitigate bias from non-random treatment assignment, we estimated treatment effects with a doubly robust augmented inverse probability (AIPW) estimator with Hájek normalisation.^43^^,^^44^

#### Qini coefficient

The normalised Qini coefficient quantifies the added benefit of targeting corticosteroid therapy based on predicted ITEs. Patients are ranked from highest to lowest benefit, partitioned into equal-sized groups, and cumulative incremental benefit is computed across groups relative to a random allocation baseline. The Qini coefficient is the area between the model's Qini curve and the baseline, normalised to an ideal benchmarking ranking to facilitate comparability across cohorts. Higher values indicate greater estimated benefits from personalised treatment.^45^^,^^46^

#### Rank-weighted average treatment benefit (RATE)

The RATE curve evaluates model ranking by estimating the average treatment effect (ATE) among fractions of the population ordered by predicted ITE. Patients are binned by rank, group-level ATEs are computed with a doubly robust Hájek AIPW estimator, and cumulative averages across bins are plotted. The overall RATE corresponds to the area under the RATE curve, reflecting the degree to which the model successfully ranks patients by true treatment benefit.^47^

#### Concordance statistic for benefit (C-for-benefit)

The C-for-benefit quantifies the model's ability to discriminate between patients with higher versus lower observed treatment effects.^48^ It estimates the probability that, for randomly selected patients, the individual with the higher predicted ITE also had a larger observed benefit. Observed effects on RMST were estimated within matched subclasses using a doubly robust AIPW estimator with Hájek normalisation. C-for-benefit values above 0·5 indicate that the model can distinguish between these patients better than random chance, with higher values indicating better discrimination.

We further assessed overall model calibration in the validation cohort. Calibration refers to the agreement between the observed and predicted treatment benefit with optimal calibration referring to both being equal.

### Sensitivity and robustness analyses

After completing model derivation, we conducted multiple sensitivity and robustness analyses to validate our findings. To quantify robustness to unmeasured confounding, we computed E-values for continuous outcomes in the validation cohort.^49^ The ATE and its standard error were standardised by the outcome standard deviation, and the point and lower-bound E-values were derived accordingly. To summarise heterogeneity, we contrasted the mean predicted ITE between the highest and lowest tertiles of predicted benefit and approximated the standard error from group-specific standard errors. E-values for this contrast were computed using the same procedure. For clinical context, we estimated the unadjusted association between baseline proteinuria and corticosteroid treatment via Poisson regression and reported the exponentiated coefficient as the risk ratio. We further assessed the estimated propensity scores for treatment assignment and compared their distributions between treated and control patients in the validation cohort. Overlap (positivity) was summarised using a kernel-based overlap coefficient, defined as the area under the minimum of the two propensity score densities. To account for any potential cohort-bias we assessed the doubly robust conditional average treatment effects across cohorts to evaluate effect modification by cohort. Additionally, the corticosteroid treatment duration and race/ethnicity as potential treatment effect modifiers were assessed similarly.

Furthermore, we added four robustness checks. (1) Random predictor: a randomly generated binary predictor was added to the derivation matrix. (2) Random replace: proteinuria was replaced by a randomly generated continuous predictor with a similar range of distribution. (3) Random treatment: the binary corticosteroid treatment assignment was changed to a random treatment assignment with similar treatment percentage. (4) Random outcome: the RMST was replaced by a random outcome with similar range to the observed RMST in the derivation cohort. For each check, the model was refit and model robustness was assessed by comparing the predicted ATE with 95% confidence intervals by bootstrapping in the derivation cohort.

### Scoring of tubulointerstitial inflammation

To further assess the importance of combining tubulointerstitial pathomics features, 60 cases with increased tubular distance were scored by a trained nephropathologist for interstitial inflammation (*i*) and tubulitis (*t*) in accordance with the Banff classification for kidney transplant pathology.^50^ Interstitial inflammation was defined as i0: no inflammation or in less than 10% of unscarred cortical parenchyma, i1: inflammation in 10–25% of unscarred cortical parenchyma, i2: inflammation in 26–50% of unscarred cortical parenchyma and i3: inflammation in more than 50% of unscarred cortical parenchyma. Tubulitis was defined as t0: no mononuclear cells in tubules or single focus of tubulitis only, t1: two or more foci with one to four mononuclear cells/tubular cross section (or ten tubular cells) in the most affected tubule, t2: two or more foci of tubulitis, at least one of those foci with five to ten mononuclear cells/tubular cross section (or ten tubular cells) in the most affected tubule and t3: two or more foci of tubulitis, at least one of those foci with over ten mononuclear cells/tubular cross section in the most affected tubule, or the presence of ≥2 areas of tubular basement membrane destruction accompanied by i2/i3 inflammation and t2 elsewhere. Each half of the cases represented cases with high or low predicted corticosteroid treatment effect, and all cases were scored in blinded fashion without any knowledge of predicted treatment benefit, associated clinical or pathomics data.

### Statistics

Patients were ranked based on their predicted individualised treatment effect, i.e., the difference in RMST with versus without corticosteroid therapy, and stratified into tertiles in accordance with the PATH statement.^25^ Within each tertile, we estimated the conditional average treatment effect (CATE) using a doubly robust, Hájek normalised AIPW estimator with propensity clipping for weight stability and uncertainty quantified via a nonparametric bootstrap with 1000 iterations. A joint Wald test was implemented to assess heterogeneity of estimated benefit across tertiles. Furthermore, we implemented a treatment recommendation for corticosteroid therapy based on the Qini curve and distribution of individualised treatment effect. Patients in the upper third of predicted individualised treatment effect (high benefit) were categorised as recommended to be treated with corticosteroids while the middle and lower tertile (low benefit) were recommended not to be treated. To assess clinical utility, we estimated the doubly robust treatment benefit by recommendation strata, with bootstrapped 95% confidence intervals and Bonferroni-adjusted p-values for multiple comparisons between groups Progression-free kidney survival was compared between groups using Kaplan–Meier curves.

Feature importance in predicting individualised treatment effects was analysed by SHAP and partial dependence analyses in the validation cohort.

Continuous variables were summarised as median (interquartile range, IQR) and categorical variables as n (%). All statistical tests were two-sided, with significance defined as *p* < 0·05. All analyses were performed within R version 4.4.3.

### Ethics

Data collection and analysis in this study was performed in accordance with the Declaration of Helsinki. The study was approved by the local ethics committee of the RWTH Aachen University (EK-No. 125/25), which covered harmonised secondary use of data across all eight cohorts. Local institutional review board or ethics committee approvals were obtained at each participating centre prior to data contribution.

Written informed consent was obtained from participants in the CureGN and NURTuRE-CKD cohorts; for all other cohorts (VALIGA, Kyoto, Leicester, Aachen, Diyarbakir and Rochester), consent was waived by the respective local ethics committees in accordance with applicable national and institutional regulations. There was no difference in consent protocol for adolescents or adults. Waivers covered all clinical, histopathological, and imaging data used in this study.

All data were de-identified prior to transfer and analysis in accordance with GDPR, and no patient-identifiable information was transferred between sites or used in any analysis.

### Role of funders

None of the funders had any role in study design, data collection, data analysis, data interpretation, or writing of the report.

## Results

### Patient characteristics and outcomes

Both the multicentric derivation (overall n = 464, VALIGA (n = 352), Kyoto (n = 92), NURTuRE-CKD (n = 20)) and validation cohorts (overall n = 558, VALIGA (n = 155), Leicester (n = 93), Aachen (n = 27), Diyarbakir (n = 106), Rochester (n = 42), CureGN (n = 135)) exhibited similar baseline characteristics and follow-up durations (Fig. 1, Table 1, Supplementary Tables S3 and S4). Notable differences were observed in the Oxford classification scores, with higher prevalence of mesangial hypercellularity (55·9% (n = 312) vs 31·9% (n = 148)), endocapillary hypercellularity (24·0% (n = 134) vs 16·2% (n = 75)), and presence of crescents (21·0% (n = 117) vs 13·6% (n = 63)) in the validation cohort, especially in the Leicester, Aachen, and CureGN cohorts, generally indicating more active disease states. Renin-angiotensin system inhibitor (RASi) usage was consistent between cohorts (80·2% (n = 372) vs 80·8% (n = 451)), whereas corticosteroid therapy was more common in the derivation cohort (34·3% (n = 159) vs 20·1% (n = 112)) and administered for a longer duration (1·1 vs 0·6 years). The primary composite outcome (≥50% eGFR decline and/or kidney failure within 5 years) occurred in 8·6% (n = 40) of the derivation cohort and 8·8% (n = 49) of the validation cohort, indicating similar event rates between cohorts (log-rank test, *p* = 0·79, Supplementary Figure S1A). At the cohort-level, corticosteroids showed no significant improvement in progression-free kidney survival (log-rank test, *p* = 0·27, Supplementary Figure S1B).

**Table 1:** Characteristics of the derivation and validation cohort.

	Derivation	Validation
n	464	558
Follow-up (years)	4·6 (6·1)	5·0 (6·0)
Age (years)	35·9 (21·1)	36·7 (20·9)
Sex: male	316 (68·1%)	364 (65·2%)
Sex: female	148 (31·9%)	194 (34·8%)
eGFR (ml/min/1·73 m^2^)	82·6 (50·1)	76·8 (52·1)
Proteinuria (g/24 h)	1·0 (1·8)	1·1 (1·6)
MAP (mmHg)	96·7 (16·7)	93·8 (17·3)
M [0|1]	0: 316 (68·1%) | 1: 148 (31·9%)	0: 246 (44·1%) | 1: 312 (55·9%)
E [0|1]	0: 389 (83·8%) | 1: 75 (16·2%)	0: 424 (76·0%) | 1: 134 (24·0%)
S [0|1]	0: 98 (21·1%) | 1: 366 (78·9%)	0: 190 (34·1%) | 1: 368 (65·9%)
T [0|1|2]	0: 369 (79·5%) | 1: 87 (18·8%) | 2: 8 (1·7%)	0: 383 (68·6%) | 1: 164 (29·4%) | 2: 11 (2·0%)
C [0|1]	0: 401 (86·4%) | 1: 63 (13·6%)	0: 441 (79·0%) | 1: 117 (21·0%)
Tuft circularity	0·36 (0·09)	0·36 (0·1)
Tuft eccentricity	0·68 (0·09)	0·67 (0·1)
Tuft area (μm^2^)	6207·01 (8548·22)	5652·7 (9686·48)
Tubular diameter (μm)	27·73 (6·73)	28·6 (6·75)
Tubular distance (μm)	2·13 (1·03)	2·26 (1·14)
RASi (y/n)	y: 372 (80·2%) | n: 92 (19·8%)	y: 451 (80·8%) | n: 107 (19·2%)
Corticosteroids (y/n)	y: 159 (34·3%) | n: 305 (65·7%)	y: 112 (20·1%) | n: 446 (79·9%)
CS therapy duration (years)	1·1 (1·1)	0·6 (0·6)
Outcome (y/n)	y: 40 (8·6%) | n: 424 (91·4%)	y: 49 (8·8%) | n: 509 (91·2%)

Continuous variables are reported as median (interquartile range) while categorical variables are reported as absolute (n) and relative (%) frequencies. The primary outcome was defined as a composite of ≥50% eGFR reduction and kidney failure within five years of kidney biopsy.

Abbreviations: m, male; f, female; eGFR, estimated glomerular filtration rate; map, mean arterial blood pressure; M, mesangial hypercellularity; E, endocapillary hypercellularity; S, segmental glomerulosclerosis; T, tubular atrophy and interstitial fibrosis; C, crescents; y, yes; n, no; RASi, renin-angiotensin-system inhibitor; CS, corticosteroid.

### Individualised treatment effects

**Fig. 2:**
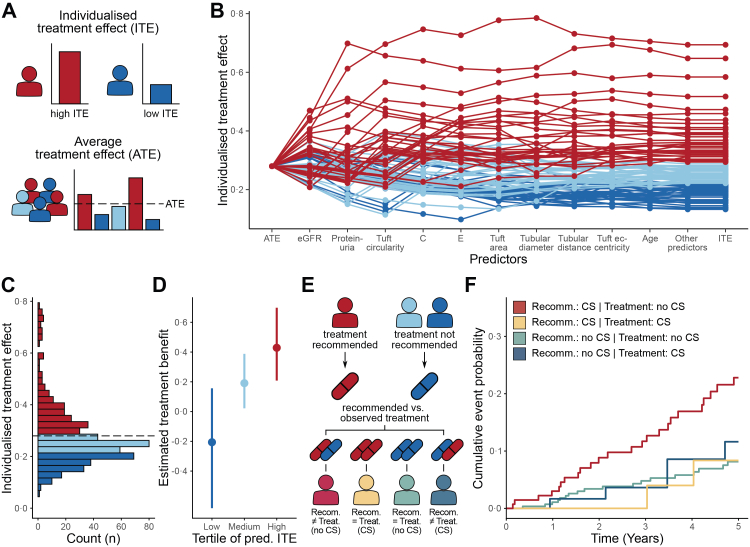
**Predicted individualised treatment effects and individual treatment recommendations. (A)** Illustration of average treatment effect and individualised treatment effect. **(B)** Line plot showing the change from the cohort-level average treatment effect to predicted patient-based individualised treatment effects based on the sequential addition of the most-important predictors. Each line represents one patient randomly chosen from each percentile from the 95% distribution of the predicted individualised treatment effect in the validation cohort (n = 100). Based on the distribution of the predicted individualised treatment effect **(C)** patients were grouped into tertiles (red (n = 186): upper tertile, high predicted benefit; light blue (n = 186): middle tertile, medium predicted benefit; blue (n = 186), lower tertile, low predicted benefit) and compared with the doubly robust estimated treatment benefit, error bars denote 95% bootstrap confidence intervals **(D)**. **(E)** Implementation of a treatment recommendation for the individuals with highest predicted benefit (red): individualised treatment recommendations were then retrospectively compared to the observed outcomes (with corticosteroid therapy vs. without corticosteroid therapy) during follow-up. **(F)** Kaplan–Meier curves comparing patients in the validation cohort (n = 558) who received the recommended treatment versus those who did not. Abbreviations: eGFR, estimated glomerular filtration rate; C, crescents; E, endocapillary hypercellularity; CS, corticosteroids.

Individualised treatment effects computed by the causal ML model revealed substantial heterogeneity in predicted treatment benefit (Fig. 2B and C). Estimated treatment benefit, defined as the difference in progression-free kidney survival, varied significantly across tertiles of predicted effect (low, middle, high; joint Wald test, *p* < 0·05 in both cohorts; Fig. 2D), with the greatest treatment benefit among patients in the upper tertile.

Based on the observed heterogeneity of treatment effects, we evaluated an individualised treatment recommendation. Considering the distribution of predicted benefit and the associated uplift gain (Supplementary Figure S2A), we assessed a model-based treatment recommendation targeting patients in the upper tertile (high predicted benefit). Patients in the middle and lower tertiles were not recommended to receive corticosteroid therapy. We retrospectively compared model-based treatment recommendations to the estimated treatment benefit (Fig. 2D and E). Among patients for whom the model recommended corticosteroid therapy, treatment was associated with longer progression-free kidney survival (derivation: 0·63 years [95% CI 0·27–0·97], Wald test, *p* < 0·01; validation: 0·43 years [95% CI 0·18–0·73], Wald test, *p* < 0·01; Fig. 2E and F). In contrast, for patients where corticosteroid therapy was not recommended by the model, the estimated benefit was not significantly different (derivation: −0·15 years [95% CI −0·33 to 0·02], Wald test, *p* > 0·05; validation: −0·005 years [95% CI −0·3 to 0·22], Wald test, *p* > 0·05; Fig. 2E and F). Implementing these model-based treatment recommendations would have reduced corticosteroid use by 67·3% and 60·7% for patients in the middle and lower tertiles of the derivation and validation cohorts, respectively. Detailed clinical characteristics of all subgroups in both cohorts are presented in Supplementary Tables S5 and S6.

### Model performance

The uplift in the Qini curve and increase in the rank-weighted average treatment effect (RATE) curve demonstrated a positive association between higher predicted individualised treatment effect and improved progression-free kidney survival (Supplementary Figure S2), indicating effective patient stratification by expected corticosteroid benefit. The Qini coefficient, i.e., the additional treatment benefit gained by targeting corticosteroid therapy using model predictions, was 0·3 (95% CI 0·15–0·42) in the derivation and 0·17 (95% CI 0·04–0·31) in the validation cohort, with higher values reflecting the improvements in patient outcomes due to personalised treatment allocation. The corresponding rank-average treatment effect (RATE) was 0·39 (95% CI 0·15–0·64) and 0·3 (95% CI 0·14–0·48), respectively. The model's ability to discriminate patients with greater or lesser treatment benefit was confirmed by a C-for-benefit of 0·59 (95% CI 0·53–0·65) in the derivation and 0·6 (95% CI 0·53–0·67) in the validation cohort.

The model was well calibrated for patients with high and medium predicted benefit, whereas predictions for low-benefit groups, i.e., the lower tertile, were overestimated (Supplementary Figure S3).

### Treatment-covariate interactions

**Fig. 3:**
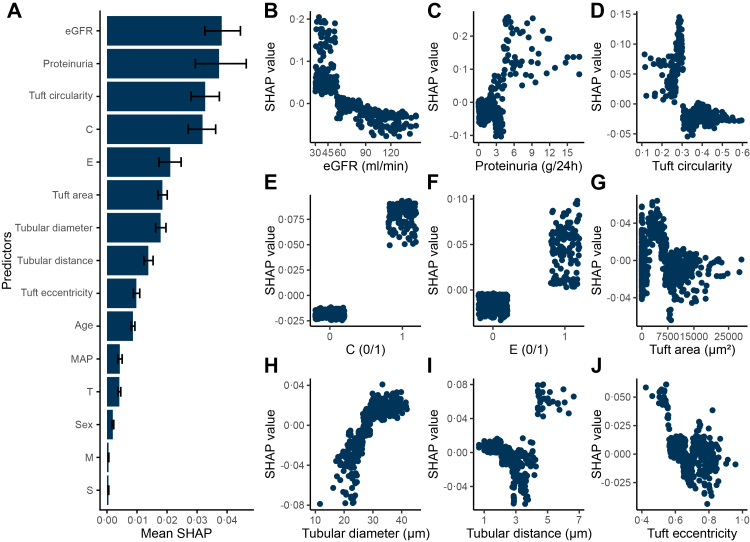
**Derived Shapley Additive Explanations (SHAP) from the validation cohort (n = 558). (A)** Aggregation of global feature importance with 95% confidence intervals (error bars) via bootstrapping with 1000 iterations and influence of the nine most important clinical, histopathological, and pathomics-based predictors on the individualised treatment effect on patient-level **(B**–**J)**. Higher SHAP values correspond to a greater predicted treatment benefit of corticosteroid therapy. Abbreviations: eGFR, estimated glomerular filtration rate; C, crescents; E, endocapillary hypercellularity.

Deconstructing model predictions by Shapley additive explanations (SHAP), identified the most influential predictors determining corticosteroid treatment benefit (Fig. 3A), including baseline eGFR, proteinuria, glomerular tuft circularity, presence of crescents (C1), endocapillary hypercellularity (E1), and tuft area. Lower baseline eGFR and higher proteinuria were most strongly associated with increased treatment benefit (Fig. 3B and C), followed by pathomics and MEST-C scores. The relative influence of the ten most important predictors on patients’ predicted treatment effect is illustrated in Fig. 2B, and representative cases of high and low predicted benefit are depicted in Supplementary Figure S4.

SHAP values of the Oxford classification components demonstrated the importance of active lesions (E1/C1) as a sign of glomerular inflammation. Progressive tubulointerstitial scarring (T1/T2) was associated with less benefit. For mesangial hypercellularity (M1) and segmental glomerulosclerosis (S1) no marked effect was detected (Supplementary Figure S5).

**Fig. 4:**
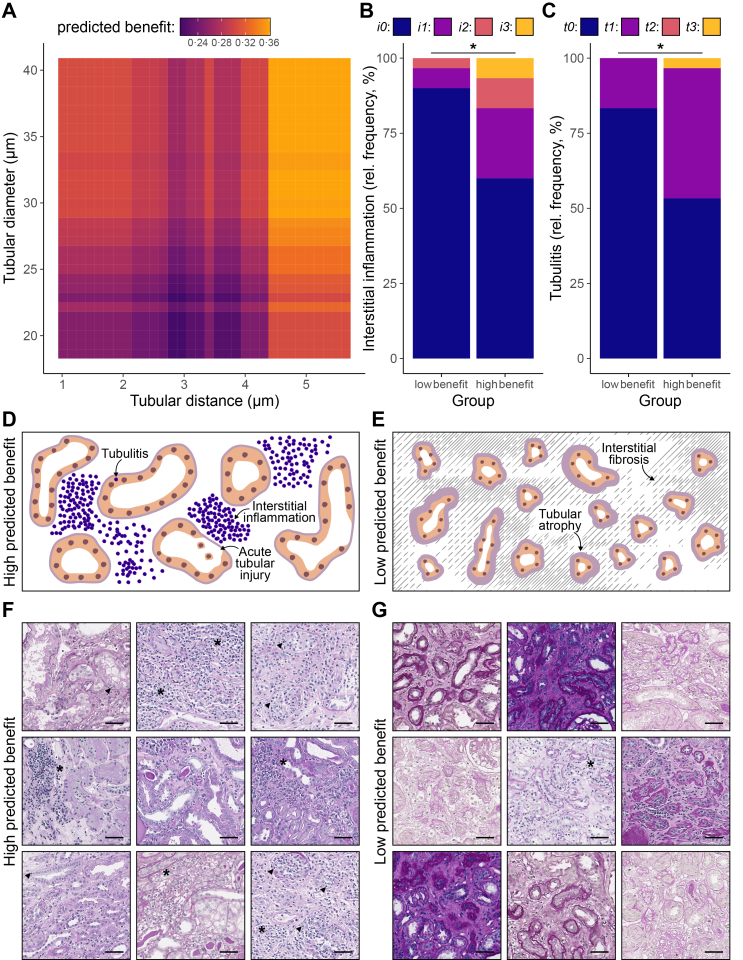
**Identification of a tubulointerstitial pathomics pattern representing tubulointerstitial inflammation. (A)** Partial dependence plot of tubular distance and tubular diameter depicting the combined effect of tubulointerstitial pathomics features on the predicted treatment benefit. Box plots for pathologist scoring demonstrated that interstitial inflammation (i, **B)** and tubulitis (t, **C)** occurred more often in representative patients with higher predicted treatment benefit (n = 30) compared to those with lower benefit (n = 30). Patients with high predicted benefit displayed more acute inflammatory IgAN disease activity **(D)** and acute tubular injury, while patients with low predicted benefit exhibited more chronic changes **(E)** such as tubular atrophy and interstitial fibrosis. **(F, G)** Representative images for cases with high and low predicted benefit. Arrows show tubulitis while stars highlight areas with increased interstitial inflammation. Scale bar: 50 μm.

Among pathomics-derived predictors, deformation of the glomerular tuft, marked by lower tuft circularity (irregular shape) and tuft eccentricity (globular shape), was associated with greater treatment benefit (Supplementary Figure S6). The tuft area exhibited a parabolic relationship with decreased benefit at the tails of the distribution, likely corresponding to sclerotic glomeruli with a small tuft area and hypertrophic glomeruli with a large tuft area (Fig. 3G). Tubular distance and diameter also ranked among the ten most influential predictors (Fig. 3A), with smaller tubular diameter and greater tubular distance (indicating tubular atrophy and interstitial fibrosis) being associated with decreased treatment benefit (Fig. 3H and I). However, patients with markedly increased tubular distance unexpectedly showed benefit from corticosteroid therapy. Partial dependence analysis revealed a combination of high tubular distance and large tubular diameter associated with high predicted benefit (Fig. 4A). Histopathological comparison of cases with this pathomics pattern (n = 30) versus those with high tubular distance and low predicted benefit (n = 30) identified a significant increase in interstitial inflammation (Wilcoxon rank-sum test, *p* < 0·05) and tubulitis (Wilcoxon rank-sum test, *p* < 0·05). Patients exhibiting this distinct pathomics pattern were four times more likely to exhibit interstitial inflammation (i1–3, Figs. 4B) and 2·8 times more likely to show tubulitis (t1–3, Fig. 4C, F and G, Supplementary Table S7).

### Sensitivity and robustness

To validate the model predictions, we conducted multiple sensitivity and robustness analyses. E-values quantify the minimum strength of association, on the risk-ratio (RR) scale, that a single unmeasured confounder would need with both treatment and outcome to reduce the estimate to the null (RR = 1). The overall RR was 1·29 (95% CI 1·28–1·3) with an E-value of 1·9 (95% CI lower bound 1·87). Across subgroups, the RR was 1·24 (95% CI 1·22–1·27) with an E-value of 1·79 (95% CI lower bound 1·73). For clinical context, baseline proteinuria showed a smaller unadjusted association (RR 1·2, 95% CI 1·08–1·34) with treatment. Propensity scores between treatment groups demonstrated sufficient overlap (Supplementary Figure S7), with an overlap coefficient of 0·88 and no propensity scores below 0·1 or above 0·9. We also analysed potential treatment-effect modifiers by comparing adjusted treatment effects across patient cohorts, race/ethnicity, and corticosteroid duration. We found no evidence that patient cohorts significantly modified the corticosteroid effect (joint Wald test, *p* = 0·24; Supplementary Figure S8). Additionally, race/ethnicity did not significantly modify the effect (joint Wald test, *p* = 0·4), although the analysis was underpowered, particularly for Chinese patients. Among treated patients, the duration of corticosteroid treatment, i.e., shorter (up to 6 months) vs. longer treatment duration (over 6 months) also did not significantly modify the treatment effect (joint Wald test, *p* = 0·36). Robustness analysis of introducing a random predictor did not substantially alter overall model performance, as the predicted average treatment effect remained consistent. In contrast, replacing proteinuria with a random continuous variable led to a notable difference in predicted average treatment effect. Moreover, randomly assigning treatment exposure or outcomes resulted in a marked decline in predicted average treatment effect, confirming the model's reliance on biologically meaningful relationships in the data (Supplementary Table S8).

## Discussion

In this retrospective, worldwide multicentre study, we developed and validated a causal ML model for predicting individualised treatment effects of systemic corticosteroid therapy in IgAN. Consistent with previous trials on the effectiveness of corticosteroid treatment,^5^^,^^51^ we could not observe a pooled significant benefit in our study. In contrast, utilising the causal ML framework, we identified a subset of patients exhibiting significant clinical benefit and those who did not. By specifically identifying patients with a high likelihood of benefiting from systemic corticosteroid therapy, usage of our model would have theoretically reduced unnecessary corticosteroid exposure by 60·7% in the validation cohort, while at the same time improving patient outcomes.

The key predictors identified are consistent with a pattern of steroid-responsive inflammation in IgAN: lower eGFR, higher proteinuria, and specific Oxford classification scores (C1, E1), which are established markers of inflammatory disease activity. Presence of crescents (C1-Score) and endocapillary hypercellularity (E1-Score) have previously been associated with response to immunosuppressive therapy.^11^^,^^52^ Patients with lower tuft circularity, a morphometric pattern suggesting glomerular tuft distortion, showed greater benefit from corticosteroid therapy. We previously showed that this feature is indicative of a more severe disease state in IgAN.^38^ Additionally, pathomics predictors reflecting tubulointerstitial changes identified a subset of patients with tubulointerstitial inflammation. This lesion was initially omitted from the Oxford classification due to poor reproducibility,^10^ yet multiple studies indicate its potential role in disease progression,^53^ although results on corticosteroid effectiveness were underpowered.^53^ In our study, pathomics enabled a precise characterisation of the tubulointerstitial architecture, the importance of which has been also demonstrated in other glomerular diseases.^54^ However, further independent validation of this patient subgroup is needed, as these findings remain exploratory and hypothesis-generating. Whether tubulointerstitial inflammation reflects IgAN disease activity or an overlapping condition, e.g., tubulointerstitial nephritis, benefiting from corticosteroid treatment remains to be further investigated. Nonetheless, reporting the degree of tubulointerstitial inflammation by pathologists could be easily implemented into current practice, e.g., by reporting the widely used Banff lesion scores *i* and *t* in IgAN biopsies.

By identifying patient-specific baseline characteristics as modifiers of corticosteroid treatment response, our approach provides an explanation as to why previous pooled analyses have failed to demonstrate consistent benefits. This paradigm shift from generic to individualised treatment decisions addresses a fundamental constraint in current IgA nephropathy management.

Our study has some limitations. The retrospective design may introduce unmeasured confounding and treatment selection bias, although a sensitivity analysis suggested that any single unmeasured confounder would need to be markedly stronger than proteinuria's association with treatment to fully nullify the estimated effect, and no positivity violations were detected. Given the framework's exploratory design and hypothesis-generating findings, prospective and independent validation in randomised controlled trials will be necessary to further establish its clinical utility. Differences in data collection standards, reporting of predictors, and treatment interventions could limit the predictive performance of our model, although a sensitivity analysis did not show any substantial influence of cohort heterogeneity. This is especially important given missing information on dosing regimens and routes of administration. Although the pathomics-derived features demonstrated predictive value, they represent an emerging exploratory approach that is not yet standardised or widely adopted in routine diagnostic workflows. Our analysis is also limited to initiation of therapy within a year after biopsy, thus, we cannot account for any longitudinal changes that might inform treatment decisions after the specified timeframe. Given the specified follow-up period of 5 years and the relatively low number of outcome events in subgroup strata, the long-term effects of treatment on disease progression remain to be investigated, potentially partly explaining the only modest improvement of progression-free kidney survival in the high benefit group and the importance of kidney function at time of biopsy. Additionally, while the model's overestimation of predicted benefit in the low-benefit group may increase the risk of over-treatment, this risk remains substantially lower than observed under the current treatment assignment in the cohort. While the current analysis focused on systemic corticosteroids and established clinical variables, the framework could be extended to other immunosuppressants, novel treatments and integrated with emerging biomarkers in the future.

Our findings reveal that apparent treatment failures at the population-level may mask significant benefits in well-defined patient subgroups, highlighting the importance of accounting for treatment effect heterogeneity in clinical research and practice. Using highly granular pathomics features might aid this effort in helping to identify specific phenotypes that might be missed on a case-to-case human evaluation. Our multimodal approach could be readily adapted to the rapidly expanding treatment landscape of IgAN as well as other glomerular diseases, where tissue-based biomarkers can guide individualised decision-making and precision nephrology.

## Contributors

Conceptualisation: DLH, RDB; Methodology: DLH; RDB; Software: DLH, NEJS, LN, PP, MS, JMJ; Validation: DLH, RDB; Formal analysis: DLH; Investigation: DLH; Resources: PB, RDB; Data curation: DLH, NEJS, LN, PP, MS, VT, JB, ISDR, RC, LB, MY, UA, ADR, ESA, CS, SS, MWT, PAK, JF, RK, PB, RDB; Data access and verification: DLH, RDB, PB; Writing–Original Draft: DLH, RDB; Writing–Review & Editing: DLH, NEJS, PP, MS VT, JB, ISDR, RC, LB, MY, UA, ADR, JMJ, ESA, CS, SS, MWT, PAK, JF, RK, PB, RDB; Visualisation: DLH; Supervision: RDB, PB; Project administration: None; Funding acquisition: PB, RDB. The VALIGA and CureGN investigators, NURTuRE academic steering group, and the AI4IgAN study members contributed to data collection and curation. All authors have read and approved the final version of the manuscript.

## Data sharing statement

Whole slide image files and clinical data used in this study are available under restricted access for privacy protection reasons. Access for research purposes can be obtained by directly contacting the corresponding author who can distribute requests to the collaborators involved who contributed data. Alternatively, to obtain access for individual datasets, all included study groups and contributing collaborators are provided in Supplementary Table S9. In general, requests will be evaluated within a timeframe of 4 weeks based on institutional and trial policies.

Annotated code on model derivation and validation as well as all subsequently performed analyses is available under GitLab (https://git-ce.rwth-aachen.de/labooratory-ai/igan-ite-prediction).

## Declaration of interests

JB reports disclosures from consulting with Alexion, AstraZeneca, Biogen, Calliditas, Novartis, Otsuka, Roche, Takeda, Travere, Vera, Vertex and research funding from Alexion, Biogen, Calliditas, Novartis, Otsuka, Travere, Vera, Vertex.

ISDR reports consultancy agreements with Alexion, Novartis, Purespring Therapeutics, Travere Therapeutics, and Vera Therapeutics, support for attending meetings and travel from Travere Therapeutics, and serves as the director UK Renal Pathology Limited.

LB reports funding for Cure Glomerulonephropathy (5U24-DK100845-10) from the National Institute of Diabetes and Digestive and Kidney Diseases (NIDDK).

MY reports funding from AMED (Platform Program for Promotion of Genome Medicine; Translational Research, Seeds A; Moonshot Research and Development Program; Project for Elucidation of the role of tertiary lymphoid tissue in human kidney disease and its potential as a target for therapeutic intervention; Leading Advanced Projects for medical innovation: LEAP), AMED-CREST (Understanding of Pathophysiological Processes and Discovery of Medical Technology Seeds through Spatiotemporal Research of Tissue Adaptation and Repair Mechanisms), Grant-in-Aid for challenging Exploratory Research and for Scientific Research (B/C), Boehringer Ingelheim GmbH, Innovate the World with University-launched Startups, payment or honoraria for lectures from Astellas Pharma Inc., AstraZeneca, Kyowa Kirin, Mitsubishi Tanabe Pharma Corporation, Chugai Pharmaceutical, and participation on a data safety monitoring board or advisory board for AstraZeneca and VIATRIS.

CS reports research funding from STADA and CSL Vifor, consulting fees from STADA and CSL Vifor, honoraria from lectures or presentations from STADA, CSL Vifor and Novartis, support for attending meetings from STADA and CSL Vifor and participation on a data safety monitoring or advisory board from STADA, CSL Vifor, Sobi and Otsuka.

MWT reports royalties or licences for Elsevier publishers for the role as co-editor of “Brenner and Rector's The Kidney” textbook, consulting fees for Boehringer Ingelheim, payment or honoraria for lectures and presentations from Bayer, Wiley publishers and Boehringer Ingelheim, support for attending meetings and travel from Bayer, and chair of iNET-CKD and deputy chair of research working group for the International Society of Nephrology (ISN) none of which was related to this work.

PAK has received honoraria for lecturing and advisory boards from Medice, Stadam Boehringer Ingelheim, Novo Nordisk, CSL Vifor and Pharmacosmos.

JF received consultancy and/or lecture honoraria from Alexion, AstraZeneca, Biogen, Boehringer, CSL Vifor, Novartis, Otsuka, Roche, Stadapharm, Vera Therapeutics and Vertex.

RK is a founder, shareholder, and board member of Sequantrix GmbH, a member of the scientific advisory board of Hybridise Therapeutics and has received honoraria for advisory boards and talks from Bayer, Chugai, Pfizer, Roche, Genentech, Lilly, and GSK and has received research funding from Travere Therapeutics, Galapagos, Novo Nordisk and Ask Bio.

PB received consulting and lecture fees from CSL Vifor, Otsuka, Novartis, Bristol Myers Squibb, Roche, Novo Nordisk, Astellas, and aetherAI none of which was relevant to this work.

The CureGN consortium is funded by U24DK100845, U01DK100846, U01DK100876, U01DK100866, and U01DK100867 from the National Institute of Diabetes and Digestive and Kidney Diseases (NIDDK).

The VALIGA study was granted by the first research call of the European Renal Association-European Dialysis and Transplant Association (ERA-EDTA) in 2009.

Funding for NURTuRE-CKD is provided and coordinated by Kidney Research UK, the University of Nottingham, UCB Biopharma, Evotec International GmbH, AstraZeneca, AbbVie and Travere Therapeutics.

Funding for the Leicester Mayer IgA Nephropathy Research Group was provided by the Mayer Family Fund.

The other authors declare no competing interests.

## Appendix A Supplementary data

Supplementary Tables and Figures

VALIGA investigators

NURTuRE investigators

AI4IgAN investigators

CureGN investigators

## References

[1] Barratt J., Feehally J. (2005). IgA nephropathy. J Am Soc Nephrol.

[2] Pitcher D., Braddon F., Hendry B. (2023). Long-term outcomes in IgA nephropathy. Clin J Am Soc Nephrol.

[3] Zhang Z., Yang Y., Jiang S.-M., Li W.-G. (2019). Efficacy and safety of immunosuppressive treatment in IgA nephropathy: a meta-analysis of randomized controlled trials. BMC Nephrol.

[4] Rovin B.H., Barratt J., Kidney Disease Improving Global Outcomes KDIGO IgAN and IgAV Work Group (2025). KDIGO 2025 clinical practice guideline for the management of immunoglobulin A nephropathy (IgAN) and immunoglobulin A vasculitis (IgAV). Kidney Int.

[5] Rauen T., Eitner F., Fitzner C. (2015). Intensive supportive care plus immunosuppression in IgA nephropathy. N Engl J Med.

[6] Lv J., Wong M.G., Hladunewich M.A. (2022). Effect of oral methylprednisolone on decline in kidney function or kidney failure in patients with IgA nephropathy: the TESTING randomized clinical trial. JAMA.

[7] Pozzi C., Bolasco P.G., Fogazzi G.B. (1999). Corticosteroids in IgA nephropathy: a randomised controlled trial. Lancet.

[8] Tesar V., Troyanov S., Bellur S. (2015). Corticosteroids in IgA nephropathy: a retrospective analysis from the VALIGA study. J Am Soc Nephrol.

[9] Cai Q., Xie X., Wang J. (2017). Severe adverse effects associated with corticosteroid treatment in patients with IgA nephropathy. Kidney Int Rep.

[10] Roberts I.S.D., Cook H.T., Working Group of the International IgA Nephropathy Network and the Renal Pathology Society (2009). The Oxford classification of IgA nephropathy: pathology definitions, correlations, and reproducibility. Kidney Int.

[11] Haas M., Verhave J.C., Liu Z.H. (2017). A multicenter study of the predictive value of crescents in IgA nephropathy. J Am Soc Nephrol.

[12] Bellur S.S., Roberts I.S.D., Troyanov S. (2019). Reproducibility of the Oxford classification of immunoglobulin A nephropathy, impact of biopsy scoring on treatment allocation and clinical relevance of disagreements: evidence from the VALidation of IGA study cohort. Nephrol Dial Transplant.

[13] Barisoni L., Lafata K.J., Hewitt S.M., Madabhushi A., Balis U.G.J. (2020). Digital pathology and computational image analysis in nephropathology. Nat Rev Nephrol.

[14] Bülow R.D., Hölscher D.L., Costa I.G., Boor P. (2023). Extending the landscape of omics technologies by pathomics. NPJ Syst Biol Appl.

[15] Perkovic V., Barratt J., Rovin B. (2025). Alternative complement pathway inhibition with iptacopan in IgA nephropathy. N Engl J Med.

[16] Mathur M., Barratt J., Chacko B. (2024). A phase 2 trial of sibeprenlimab in patients with IgA nephropathy. N Engl J Med.

[17] Coppo R., Troyanov S., Bellur S. (2014). Validation of the Oxford classification of IgA nephropathy in cohorts with different presentations and treatments. Kidney Int.

[18] Mariani L.H., Bomback A.S., Canetta P.A. (2019). CureGN study rationale, design, and methods: establishing a large prospective observational study of glomerular disease. Am J Kidney Dis.

[19] Taal M.W., Lucas B., Roderick P. (2023). Associations with age and glomerular filtration rate in a referred population with chronic kidney disease: methods and baseline data from a UK multicentre cohort study (NURTuRE-CKD). Nephrol Dial Transplant.

[20] Trimarchi H., Barratt J., Cattran D.C. (2017). Oxford classification of IgA nephropathy 2016: an update from the IgA nephropathy classification working group. Kidney Int.

[21] I Inker L.A., Eneanya N.D., Coresh J. (2021). New creatinine- and cystatin C-based equations to estimate GFR without race. N Engl J Med.

[22] Kim D., Neuen B.L., Lv J. (2025). Corticosteroid effects in IgA nephropathy by baseline proteinuria and estimated GFR. Kidney Int Rep.

[23] Denic A., Bogojevic M., Mullan A.F. (2022). Prognostic implications of a morphometric evaluation for chronic changes on all diagnostic native kidney biopsies. J Am Soc Nephrol.

[24] Feuerriegel S., Frauen D., Melnychuk V. (2024). Causal machine learning for predicting treatment outcomes. Nat Med.

[25] Kent D.M., Paulus J.K., van Klaveren D. (2020). The Predictive Approaches to treatment effect Heterogeneity (PATH) statement. Ann Intern Med.

[26] Collins G.S., Moons K.G.M., Dhiman P. (2024). TRIPOD+AI statement: updated guidance for reporting clinical prediction models that use regression or machine learning methods. BMJ.

[27] Betensky R.A. (2015). Measures of follow-up in time-to-event studies: why provide them and what should they be?. Clin Trials.

[28] Klein J.P., Gerster M., Andersen P.K., Tarima S., Perme M.P. (2008). SAS and R functions to compute pseudo-values for censored data regression. Comput Methods Programs Biomed.

[29] Ni A., Lin Z., Lu B. (2021). Stratified restricted mean survival time model for marginal causal effect in observational survival data. Ann Epidemiol.

[30] Schwartz G.J., Muñoz A., Schneider M.F. (2009). New equations to estimate GFR in children with CKD. J Am Soc Nephrol.

[31] Hogan M.C., Reich H.N., Nelson P.J. (2016). The relatively poor correlation between random and 24-hour urine protein excretion in patients with biopsy-proven glomerular diseases. Kidney Int.

[32] Liu X., Zhao Y., Feng Y. (2022). Estimation of 24-h urine protein excretion using urine albumin-to-creatinine ratio from an in-hospital population. Med Sci Monit.

[33] NHS data model and dictionary. https://www.datadictionary.nhs.uk/.

[34] Flanagin A., Frey T., Christiansen S.L., AMA Manual of Style Committee (2021). Updated guidance on the reporting of race and ethnicity in medical and science journals. JAMA.

[35] Yeo S.C., Goh S.M., Barratt J. (2019). Is immunoglobulin A nephropathy different in different ethnic populations?. Nephrology (Carlton).

[36] Cattran D.C., Coppo R., Working Group of the International IgA Nephropathy Network and the Renal Pathology Society (2009). The Oxford classification of IgA nephropathy: rationale, clinicopathological correlations, and classification. Kidney Int.

[37] Zhang Z. (2016). Multiple imputation with multivariate imputation by chained equation (MICE) package. Ann Transl Med.

[38] Hölscher D.L., Bouteldja N., Joodaki M. (2023). Next-generation morphometry for pathomics-data mining in histopathology. Nat Commun.

[39] Roy S., Koehler G., Ulrich C. (2023). Lecture Notes in Computer Science.

[40] Hölscher D.L., Goedertier M., Klinkhammer B.M. (2024). tRigon: an R package and Shiny App for integrative (path-)omics data analysis. BMC Bioinformatics.

[41] Künzel S.R., Sekhon J.S., Bickel P.J., Yu B. (2019). Metalearners for estimating heterogeneous treatment effects using machine learning. Proc Natl Acad Sci U S A.

[42] Nie X., Wager S. (2021). Quasi-oracle estimation of heterogeneous treatment effects. Biometrika.

[43] Lunceford J.K., Davidian M. (2004). Stratification and weighting via the propensity score in estimation of causal treatment effects: a comparative study. Stat Med.

[44] Hajek J. (1981).

[45] Belbahri M., Murua A., Gandouet O., Nia V.P. (2019).

[46] Belbahri M., Murua A., Gandouet O., Partovi Nia V. (2021). Qini-based uplift regression. Ann Appl Stat.

[47] Athey S., Tibshirani J., Wager S. (2019). Generalized random forests. Ann Stat.

[48] Maas C.C.H.M., Kent D.M., Hughes M.C., Dekker R., Lingsma H.F., van Klaveren D. (2023). Performance metrics for models designed to predict treatment effect. BMC Med Res Methodol.

[49] Mathur M.B., Smith L.H., Yoshida K., Ding P., VanderWeele T.J. (2022). E-values for effect heterogeneity and approximations for causal interaction. Int J Epidemiol.

[50] Naesens M., Roufosse C., Haas M. (2024). The Banff 2022 kidney meeting report: reappraisal of microvascular inflammation and the role of biopsy-based transcript diagnostics. Am J Transplant.

[51] Rivedal M., Haaskjold Y.L., Eikrem Ø., Bjørneklett R., Marti H.P., Knoop T. (2024). Use of corticosteroids in Norwegian patients with immunoglobulin a nephropathy progressing to end-stage kidney disease: a retrospective cohort study. BMC Nephrol.

[52] Chakera A., MacEwen C., Bellur S.S., Chompuk L.O., Lunn D., Roberts I.S.D. (2016). Prognostic value of endocapillary hypercellularity in IgA nephropathy patients with no immunosuppression. J Nephrol.

[53] Rankin A.J., Kipgen D., Geddes C.C. (2019). Assessment of active tubulointerstitial nephritis in non-scarred renal cortex improves prediction of renal outcomes in patients with IgA nephropathy. Clin Kidney J.

[54] Fan F., Liu Q., Zee J. (2025). Clinical relevance of computationally derived tubular features and their spatial relationships with the interstitial microenvironment in minimal change disease/focal segmental glomerulosclerosis. Kidney Int.

