# Supplementary material for: Individualised treatment effects of corticosteroids in IgA nephropathy
Source: eBioMedicine. 2026 Jul 14;130:106396. doi: 10.1016/j.ebiom.2026.106396 (PMC13377490; doi:10.1016/j.ebiom.2026.106396)

Supplementary appendix to:

Individualised treatment effects of corticosteroids in IgA nephropathy

David L. Hölscher, MD^1,2^, Nikolas E. J. Schmitz, MSc^1^, Leon Niggemeier, MSc^1^, Pourya Pilva, MSc^1^, Martin Strauch, PhD^1^, Vladimir Tesar, PhD^3^, Jonathan Barratt, PhD^4,5^, Ian S. D. Roberts, PhD^6^, Rosanna Coppo, MD^7,8^, Laura Barisoni, MD^9,10^, Motoko Yanagita, PhD^11,12^, Ulas Alabalik, MD^13^, Andrew D. Rule, MD^14,15^, Jaidip M. Jagtap, PhD^14^, Eliabe S. Abreu, MD^14^, Claudia Seikrit, MD^2^, Saskia von Stillfried, MD^1^, Maarten W. Taal, MD^16,17^, Philip A. Kalra, MD^18^, Juergen Floege, MD^2,19^, Rafael Kramann, PhD^2^, Peter Boor, PhD^1,2,§,*^ for the AI4IgAN study, Roman D. Bülow, MD^1,§^ on behalf of the VALIGA and CureGN investigators, the NURTuRE academic steering group, and the AI4IgAN study group

1 Institute of Pathology, RWTH Aachen University Hospital, Aachen, Germany

2 Department of Nephrology and Immunology, RWTH Aachen University Hospital, Aachen, Germany

3 Department of Nephrology, 1^st^ Faculty of Medicine and General University Hospital, Charles University, Prague, Czech Republic

4 John Walls Renal Unit, University Hospital of Leicester National Health Service Trust, Leicester, United Kingdom

5 Department of Cardiovascular Sciences, University of Leicester, Leicester, United Kingdom

6 Department of Cellular Pathology, Oxford University Hospitals National Health Services Foundation Trust, Oxford, United Kingdom

7 Fondazione Ricerca Molinette, Torino, Italy

8 Regina Margherita Children's University Hospital, Torino, Italy

9 Department of Pathology, Division of AI & Computational Pathology, Duke University, Durham, USA

10 Department of Medicine, Division of Nephrology, Duke University, Durham, USA

11 Department of Nephrology, Graduate School of Medicine, Kyoto University, Kyoto, Japan

12 Institute for the Advanced Study of Human Biology (WPI-ASHBi), Kyoto University, Kyoto, Japan

13 Department of Pathology, Medical Faculty, Dicle University, Diyarbakir, Turkey

14 Division of Nephrology and Hypertension, Mayo Clinic, Rochester, Minnesota, USA

15 Division of Epidemiology, Mayo Clinic, Rochester, Minnesota, USA

16 Centre for Kidney Research and Innovation, University of Nottingham, Derby, United Kingdom

17 Renal Unit, University Hospitals of Derby and Burton NHS Foundation Trust, Derby, United Kingdom

18 Renal Services, Salford Royal Hospital, Northern Care Alliance NHS Foundation Trust, Salford, United Kingdom

19 Department for Cardiology, RWTH Aachen University Hospital, Aachen, Germany

§ Shared senior authors

* Address correspondence to:

Peter Boor, MD, PhD

Institute of Pathology

RWTH Aachen University Hospital

Pauwelsstrasse 30

52074 Aachen, Germany

Phone: +49 241 80 85227

Table of Contents

Supp. Table 1: Missingness of predictors in derivation and validation cohort

Supp. Table 2: Definitions for pathomics features

Supp. Table 3: Additional baseline characteristics

Supp. Table 4: Baseline patient characteristics in the individual cohorts

Supp. Table 5: Derivation cohort: characteristics of partitioned subgroups

Supp. Table 6: Validation cohort: characteristics of partitioned subgroups

Supp. Table 7: Scoring of tubulointerstitial inflammation

Supp. Table 8: Robustness analyses

Supp. Table 10: Overview of datasets, study groups and contributors

Supp. Figure 1: Baseline cumulative event probability and overall treatment effect

Supp. Figure 2: Qini and rank-weighted average treatment effect

Supp. Figure 3: Model calibration

Supp. Figure 4: SHAP values of representative cases

Supp. Figure 5: SHAP values of MEST-C predictors

Supp. Figure 6: Representative visualisations of glomeruli in patients with high and low predicted treatment benefit

Supp. Figure 7: Propensity score overlap by treatment group in the validation cohort

Supp. Figure 8: Distribution of ITE within cohorts and subcohorts

Supplementary Tables and Figures

**Supplementary Table 1.** Missingness of patient characteristics in derivation and validation cohort in absolute (n) and relative (%) frequencies.

Abbreviations: M, mesangial hypercellularity; E, endocapillary hypercellularity; S, segmental glomerulosclerosis; T, tubular atrophy and interstitial fibrosis; C, crescents; RASi, renin-angiotensin-system inhibitor; CS, corticosteroid.

|  | **Derivation** | **Validation** |
| --- | --- | --- |
| n | 464 | 558 |
| Follow-up | 0 (0%) | 0 (0%) |
| Age | 0 (0%) | 0 (0%) |
| Sex | 0 0%) | 0 0%) |
| Race and ethnicity | 5 (1·08%) | 68 (12·19%) |
| BMI | 64 (13·79%) | 189 (33·87%) |
| eGFR | 0 (0%) | 0 (0%) |
| Proteinuria | 14 (3·02%) | 35 (6·27%) |
| MAP | 2 (0·43%) | 163 (29·21%) |
| M | 0 (0%) | 0 (0%) |
| E | 0 (0%) | 0 (0%) |
| S | 0 0%) | 0 0%) |
| T | 0 (0%) | 0 (0%) |
| C | 0 (0%) | 0 (0%) |
| Tuft circularity | 0 (0%) | 0 (0%) |
| Tuft eccentricity | 0 (0%) | 0 (0%) |
| Tuft area | 0 0%) | 0 0%) |
| Tubular Diameter | 0 (0%) | 0 (0%) |
| Tubular Distance | 0 (0%) | 0 (0%) |
| RASi | 0 (0%) | 0 (0%) |
| CS | 0 (0%) | 0 (0%) |
| CS therapy duration | 20 (4·31%) | 140 (25·09%) |
| Outcome | 0 (0%) | 0 (0%) |

**Supplementary Table 2.** Definitions for the five included pathomics features. All five pathomics features are summarised on patient-level using the medians of the feature distribution.

| **Feature** | **Definition** |
| --- | --- |
| Tuft area | Cross-sectional area of the glomerular tuft area [µm²] |
| Tuft circularity | Circularity of the glomerular tuft area. The circularity equals 1 for a circle.  $C= \frac{4 \times\pi\times A}{P^{2}}$; with A = area, P = perimeter |
| Tuft eccentricity | Ratio of the distance between the glomerular tuft’s focal points over the major axis’ length. The eccentricity equals 0 for a circle and 1 for ellipses.  $Ecc=\sqrt{\frac{(major axis {length}^{2} - minor axis {length}^{2})}{major axis length}}$ |
| Tubular distance | Closest distance from the border of a tubule to its neighbouring structure [µm] |
| Tubular diameter | Diameter of the largest circle fully fitting inside the tubule [µm] |

**Supplementary Table 3.** Additional baseline characteristics of the derivation and validation cohort. Continuous variables are reported as median (interquartile range) while categorical variables are reported as absolute (n) and relative (%) frequencies.

|  | **Derivation** | **Validation** |
| --- | --- | --- |
| n | 464 | 558 |
| Systolic blood pressure [mmHg] | 130·0 (20·0) | 122·5 (27·0) |
| Diastolic blood pressure [mmHg] | 80·0 (19·0) | 78·0 (16·0) |
| BMI [kg/m²] | 24·3 (5·6) | 25·7 (6·6) |
| Race and ethnicity | White = 348 (75·8%)  Black = 1 (0·2%)  South Asian = 13 (2·8%)  Chinese = 0 (0·0%)  Japanese = 92 (20·1%)  Other = 5 (1·1%) | White = 446 (91·0%)  Black = 5 (1·0%)  South Asian = 27 (5·5%)  Chinese = 4 (0·8%)  Japanese = 0 (0·0%)  Other = 8 (1·7%) |

**Supplementary Table 4.** Baseline patient characteristics in the individual cohorts. Continuous variables are reported as median (interquartile range) while categorical variables are reported as absolute (n) and relative (%) frequencies.

Abbreviations: m, male; f, female; eGFR, estimated glomerular filtration rate; MAP, mean arterial blood pressure; M, mesangial hypercellularity; E, endocapillary hypercellularity; S, segmental glomerulosclerosis; T, tubular atrophy and interstitial fibrosis; C, crescents; RASi, renin-angiotensin-system inhibitor; CS, corticosteroid.

|  | **VALIGA** | **Kyoto** | **NURTuRE-CKD** | **Leicester** | **Aachen** | **Diyarbakir** | **Rochester** | **CureGN** |
| --- | --- | --- | --- | --- | --- | --- | --- | --- |
| n | 507 | 92 | 20 | 93 | 27 | 106 | 42 | 135 |
| Follow-up [years] | 51  (6·3) | 2·5  (2·9) | 4·1  (1·4) | 7·7  (5·6) | 1·3  (3·3) | 1·7  (2·6) | 13·9  (9·3) | 6·3  (2·8) |
| Age [years] | 34·5 (20·8) | 40·2 (22·4) | 50·0  (16·3) | 39·6  (19·8) | 34·0  (25·5) | 33·0  (16·8) | 42·0  (20·5) | 38·3  (19·7) |
| Sex [m\|f] | m: 381 (75·1%) \| f: 126 (24·9%) | m: 39 (42·4%) \| f: 53 (57·6%) | m: 13 (65·0%)  \| f: 7  (35·0%) | m: 60 (64·5%)  \| f: 33  (35·5%) | m: 19 (70·4%)  \| f: 8  (29·6%) | m: 54 (50·9%)  \| f: 52  (49·1%) | m: 29  (69·0%)  \| f: 13  (31·0%) | m: 85 (63·0%)  \| f: 50  (37·0%) |
| eGFR [ml/  min/1.73m²] | 81·4 (46·5) | 97·5 (48·8) | 46·6  (15·3) | 71·5  (51·5) | 71·0  (33·6) | 86·8  (66·5) | 73·1  (33·7) | 66·0 (53·4) |
| Proteinuria [g/24h] | 1·0  (1·5) | 0·6  (1·1) | 1·2  (1·3) | 0·8  (1·2) | 1·0  (0·9) | 2·0  (3·3) | 1·3  (1·6) | 1·2  (1·6) |
| MAP [mmHg] | 96·7 (16·7) | 90·2 (15·1) | 100·0  (19·1) | 95·3  (20·0) | 92·0  (16·0) | 88·5  (13·3) | 99·5  (22·5) | 94·7 (15·0) |
| M [0\|1] | 0: 369 (72·8%) \| 1: 138 (27·2%) | 0: 50 (54·3%) \| 1: 42 (45·7%) | 0: 7  (35·0%)  \| 1: 13  (65·0%) | 0: 32  (34·4%)  \| 1: 61  (65·6%) | 0: 18  (66·7%)  \| 1: 9  (33·3%) | 0: 72  (67·9%)  \| 1: 34  (32·1%) | 0: 3  (7·1%)  \| 1: 39  (92·9%) | 0: 11  (8·1%)  \| 1: 124  (91·9%) |
| E [0\|1] | 0: 454 (89·5%) \| 1: 53 (10·5%) | 0: 68 (73·9%) \| 1: 24 (26·1%) | 0: 9  (45·0%)  \| 1: 11  (55·0%) | 0: 68  (73·1%)  \| 1: 25  (26·9%) | 0: 18  (66·7%)  \| 1: 9  (33·3%) | 0: 87  (82·1%)  \| 1: 19  (17·9%) | 0: 38  (90·5%)  \| 1: 4  (9·5%) | 0: 71  (52·6%)  \| 1: 64  (47·4%) |
| S [0\|1] | 0: 138 (27·2%) \| 1: 369 (72·8%) | 0: 6 (6·5%)  \| 1: 86 (93·5%) | 0: 5  (25·0%)  \| 1: 15  (75·0%) | 0: 21  (22·6%)  \| 1: 72  (77·4%) | 0: 6  (22·2%)  \| 1: 21  (77·8%) | 0: 91  (85·8%)  \| 1: 15  (14·2%) | 0: 17  (40·5%)  \| 1: 25  (59·5%) | 0: 4  (3·0%)  \| 1: 131  (97·0%) |
| T [0\|1\|2] | 0: 416 (82·0%) \| 1: 84 (16·6%) \| 2: 7 (1·4%) | 0: 67 (72·8%) \| 1: 24 (26·1%) \| 2: 1 (1·1%) | 0: 15 (75·0%)  \| 1: 4 (20·0%)  \| 2: 1  (5·0%) | 0: 84 (90·3%)  \| 1: 8 (8·6%)  \| 2: 1  (1·1%) | 0: 24 (88·9%)  \| 1: 2 (7·4%)  \| 2: 1  (3·7%) | 0: 37 (34·9%)  \| 1: 64 (60·4%)  \| 2: 5  (4·7%) | 0: 34  (80·9%)  \| 1: 7  (16·7%)  \| 2: 1  (2·4%) | 0: 75 (55·5%)  \| 1: 58 (43·0%)  \| 2: 1  (1·5%) |
| C [0\|1] | 0: 456 (89·9%) \| 1: 51 (10·1%) | 0: 68 (73·9%) \| 1: 24 (26·1%) | 0: 13 (65·0%)  \| 1: 7 (35·0%) | 0: 65 (69·9%)  \| 1: 28 (30·1%) | 0: 23 (85·2%)  \| 1: 4 (14·8%) | 0: 100 (94·3%)  \| 1: 6  (5·7%) | 0: 35  (83·3%)  \| 1: 7  (16·7%) | 0: 82 (60·7%)  \| 1: 53 (39·3%) |

**Supplementary Table 5.** Derivation cohort: characteristics of partitioned subgroups. Continuous variables are reported as median (interquartile range) while categorical variables are reported as absolute (n) and relative (%) frequencies. The primary outcome was defined as a composite of ≥ 50% eGFR reduction and kidney failure within five years of kidney biopsy.

Abbreviations: R, recommended; T, treated; CS, corticosteroid; m, male; f, female; eGFR, estimated glomerular filtration rate; MAP, mean arterial blood pressure; M, mesangial hypercellularity; E, endocapillary hypercellularity; S, segmental glomerulosclerosis; T, tubular atrophy and interstitial fibrosis; C, crescents; RASi, renin-angiotensin-system inhibitor.

|  | **R = no CS \|**  **T = no CS** | **R = no CS \|**  **T = CS** | **R = CS \|**  **T = CS** | **R = CS \|**  **T = no CS** |
| --- | --- | --- | --- | --- |
| n | 202 | 107 | 52 | 103 |
| Follow-up [years] | 6·0 (7·5) | 3·1 (3·7) | 3·8 (2·7) | 4·6 (6·9) |
| Age [years] | 33·1 (23·0) | 36·7 (18·5) | 39·8 (21·2) | 37·0 (20·2) |
| Sex [m\|f] | m: 136 (67·3%) \| f: 66 (32·7%) | m: 63 (58·9%) \| f: 44 (41·1%) | m: 33 (63·5%) \| f: 19 (36·5%) | m: 84 (81·6%) \| f: 19 (18·4%) |
| eGFR [ml/min/1.73m²] | 92·2 (45·6) | 89·8 (47·0) | 71·4 (47·8) | 61·0 (36·7) |
| Proteinuria [g/24h] | 0·8 (1·2) | 1·1 (1·1) | 1·7 (3·0) | 1·7 (2·6) |
| MAP [mmHg] | 95·3 (14·8) | 95·0 (16·7) | 95·5 (19·2) | 100·0 (18·7) |
| M [0\|1] | 0: 155 (76·7%) \| 1: 47 (23·3%) | 0: 67 (62·6%) \| 1: 40 (37·4%) | 0: 34 (65·4%) \| 1: 18 (34·6%) | 0: 60 (58·3%) \| 1: 43 (41·7%) |
| E [0\|1] | 0: 188 (93·1%) \| 1: 14 (6·9%) | 0: 96 (89·7%) \| 1: 11 (10·3%) | 0: 33 (63·5%) \| 1: 19 (36·5%) | 0: 72 (69·9%) \| 1: 31 (30·1%) |
| S [0\|1] | 0: 65 (32·2%) \| 1: 137 (67·8%) | 0: 16 (15·0%) \| 1: 91 (85·0%) | 0: 3 (5·8%) \| 1: 49 (94·2%) | 0: 14 (13·6%) \| 1: 89 (86·4%) |
| T [0\|1\|2] | 0: 170 (84·1%) \| 1: 28 (13·9%) \| 2: 4 (2·0%) | 0: 84 (78·5%) \| 1: 22 (20·6%) \| 2: 1 (0·9%) | 0: 37 (71·2%) \| 1: 15 (28·8%) \| 2: 0 (0·0%) | 0: 78 (75·7%) \| 1: 22 (21·4%) \| 2: 3 (2·9%) |
| C [0\|1] | 0: 198 (98·0%) \| 1: 4 (2·0%) | 0: 100 (93·5%) \| 1: 7 (6·5%) | 0: 29 (55·8%) \| 1: 23 (44·2%) | 0: 74 (71·8%) \| 1: 29 (28·2%) |
| Tuft circularity | 0·39 (0·08) | 0·36 (0·08) | 0·31 (0·07) | 0·35 (0·11) |
| Tuft eccentricity | 0·66 (0·08) | 0·69 (0·11) | 0·72 (0·13) | 0·67 (0·1) |
| Tuft area [µm²] | 7743·75 (6038·8) | 3021·07 (7340·02) | 2480·11 (5407·37) | 5554·99 (6968·55) |
| Tubular diameter [µm] | 28·06 (6·24) | 27·49 (6·1) | 26·71 (7·38) | 28·62 (6·39) |
| Tubular distance [µm] | 1·97 (1·12) | 2·38 (0·9) | 2·32 (1·1) | 2·26 (1·26) |
| RASi [y/n] | y: 159 (78·7%) \| n: 43 (21·3%) | y: 77 (72·0%) \| n: 30 (28·0%) | y: 42 (80·8%) \| n: 10 (19·2%) | y: 94 (91·3%) \| n: 9 (8·7%) |
| Corticosteroids [y/n] | y: 0 (0·0%) \| n: 202 (100·0%) | y: 107 (100·0%) \| n: 0 (0·0%) | y: 52 (100·0%) \| n: 0 (0·0%) | y: 0 (0·0%) \| n: 103 (100·0%) |
| CS therapy duration [years] | 0·0 (0·0) | 1·1 (1·1) | 1·3 (1·5) | 0 (0·0) |
| Outcome [y/n] | y: 4 (2·0%) \| n: 198 (98·0%) | y: 7 (6·5%) \| n: 100 (93·5%) | y: 3 (5·8%) \| n: 49 (94·2%) | y: 26 (25·2%) \| n: 77 (74·8%) |

**Supplementary Table 6.** Validation cohort: characteristics of partitioned subgroups. Continuous variables are reported as median (interquartile range) while categorical variables are reported as absolute (n) and relative (%) frequencies. The primary outcome was defined as a composite of ≥ 50% eGFR reduction and kidney failure within five years of kidney biopsy.

Abbreviations: R, recommended; T, treated; CS, corticosteroid; m, male; f, female; eGFR, estimated glomerular filtration rate; MAP, mean arterial blood pressure; M, mesangial hypercellularity; E, endocapillary hypercellularity; S, segmental glomerulosclerosis; T, tubular atrophy and interstitial fibrosis; C, crescents; RASi, renin-angiotensin-system inhibitor.

|  | **R = no CS \|**  **T = no CS** | **R = no CS \|**  **T = CS** | **R = CS \|**  **T = CS** | **R = CS \|**  **T = no CS** |
| --- | --- | --- | --- | --- |
| n | 304 | 68 | 44 | 142 |
| Follow-up [years] | 5·1 (6·2) | 4·5 (5·6) | 4·8 (7·4) | 4·9 (5·6) |
| Age [years] | 37·5 (20·0) | 34·2 (22·5) | 30·8 (22·8) | 37·3 (20·6) |
| Sex [m\|f] | m: 205 (67·4%) \| f: 99 (32·6%) | m: 43 (63·2%) \| f: 25 (36·8%) | m: 27 (61·4%) \| f: 17 (38·6%) | m: 89 (62·7%) \| f: 53 (37·3%) |
| eGFR [ml/min/1·73m²] | 80·5 (46·0) | 86·5 (50·5) | 60·7 (58·3) | 59·9 (50·6) |
| Proteinuria [g/24h] | 0·9 (1·1) | 1·2 (1·3) | 2·4 (4·1) | 1·6 (3·0) |
| MAP [mmHg] | 93·3 (17·8) | 95·5 (20·0) | 93·3 (19·6) | 94·8 (16·7) |
| M [0\|1] | 0: 132 (43·4%) \| 1: 172 (56·6%) | 0: 41 (60·3%) \| 1: 27 (39·7%) | 0: 15 (34·1%) \| 1: 29 (65·9%) | 0: 58 (40·9%) \| 1: 84 (59·2%) |
| E [0\|1] | 0: 260 (85·5%) \| 1: 44 (14·5%) | 0: 56 (82·4%) \| 1: 12 (17·6%) | 0: 27 (61·4%) \| 1: 17 (38·6%) | 0: 81 (57·0%) \| 1: 61 (43·0%) |
| S [0\|1] | 0: 111 (36·5%) \| 1: 193 (63·5%) | 0: 26 (38·2%) \| 1: 42 (61·8%) | 0: 13 (29·6%) \| 1: 31 (70·4%) | 0: 40 (28·2%) \| 1: 102 (71·8%) |
| T [0\|1\|2] | 0: 230 (75·7%) \| 1: 69 (22·7%) \| 2: 5 (1·6%) | 0: 40 (58·8%) \| 1: 26 (38·2%) \| 2: 2 (3·0%) | 0: 20 (45·5%) \| 1: 22 (50·0%) \| 2: 2 (4·5%) | 0: 93 (65·5%) \| 1: 47 (33·1%) \| 2: 2 (1·4%) |
| C [0\|1] | 0: 285 (93·8%) \| 1: 19 (6·2%) | 0: 58 (85·3%) \| 1: 10 (14·7%) | 0: 22 (50·0%) \| 1: 22 (50·0%) | 0: 76 (53·5%) \| 1: 66 (46·5%) |
| Tuft circularity | 0·39 (0·08) | 0·35 (0·09) | 0·30 (0·09) | 0·32 (0·09) |
| Tuft eccentricity | 0·66 (0·09) | 0·66 (0·12) | 0·68 (0·11) | 0·68 (0·13) |
| Tuft area [µm²] | 7492·56 (9568·65) | 5385·62 (9413·43) | 4358·35 (6479·06) | 2703·81 (6817·52) |
| Tubular diameter [µm] | 28·63 (7·16) | 28·59 (6·37) | 29·09 (4·48) | 28·12 (7·09) |
| Tubular distance [µm] | 2·16 (1·0) | 2·31 (1·21) | 2·68 (1·8) | 2·26 (1·04) |
| RASi [y/n] | y: 241 (79·3%) \| n: 63 (20·7%) | y: 53 (77·9%) \| n: 15 (22.1%) | y: 36 (81·8%) \| n: 8 (18·2%) | y: 121 (85·2%) \| n: 21 (14·8%) |
| Corticosteroids [y/n] | y: 0 (0·0%) \| n: 304 (100·0%) | y: 68 (100·0%) \| n: 0 (0·0%) | y: 44 (100·0%) \| n: 0 (0·0%) | y: 0 (0·0%) \| n: 142 (100·0%) |
| CS therapy duration [years] | 0·0 (0·0) | 0·5 (0·7) | 0·8 (0·5) | 0 (0·0) |
| Outcome [y/n] | y: 18 (5·9%) \| n: 286 (94·1%) | y: 5 (7·4%) \| n: 63 (92·6%) | y: 2 (4·5%) \| n: 42 (95·5%) | y: 24 (16·9%) \| n: 118 (83·1%) |

**Supplementary Table 7.** Scoring of tubulointerstitial inflammation. 60 cases with increased tubular distance (30 with high predicted benefit suggestive for active tubulointerstitial inflammation and 30 with low predicted benefit suggestive for tubulointerstitial scarring) were scored blinded in accordance with the Banff classification for kidney transplant pathology.

Abbreviations: ITE, individualised treatment effect; i, interstitial inflammation; t, tubulitis.

|  | **Low predicted ITE** | **High predicted ITE** |
| --- | --- | --- |
| Overall n | 30 | 30 |
| **Interstitial inflammation (i)** | | |
| i0 | 27 (90·0%) | 18 (60·0%) |
| i1 | 2 (6·67%) | 7 (23·33) |
| i2 | 1 (3·33%) | 3 (10·0%) |
| i3 | 0 (0%) | 2 (6·67%) |
| **Tubulitis (t)** | | |
| t0 | 25 (83·33%) | 16 (53·33%) |
| t1 | 5 (16·67%) | 13 (43·33%) |
| t2 | 0 (0%) | 0 (0%) |
| t3 | 0 (0% | 1 (3·33%) |

**Supplementary Table 8.** Robustness analyses comparing the predicted average treatment effect of the fitted model to random perturbations of covariates, treatment and outcome.

| **Complete model** | |
| --- | --- |
| Average treatment effect | 0·254 (95% CI 0·244-0·268) |
| **Random predictor** | |
| Average treatment effect | 0·236 (95% CI 0·224-0·25) |
| **Random replace** | |
| Average treatment effect | 0·227 (95% CI 0·209-0·251) |
| **Random treatment** | |
| Average treatment effect | -0·05 (95% CI -0·071-(-0·029)) |
| **Random outcome** | |
| Average treatment effect | 0·094 (95% CI 0·068-0·119) |

**Supplementary Table 9.** Overview of datasets, study groups and contributors**.**

| **Dataset** | **Contact** | **Contact** |
| --- | --- | --- |
| VALIGA | Rosanna Coppo for the VALIGA investigators | Email: copporosanna50@gmail.com |
| Kyoto | Motoko Yanagita | Email: motoy@kuhp.kyoto-u.ac.jp |
| NURTuRE-CKD | Maarten W. Taal & Philip A. Kalra for the NURTuRE academic steering group | Email: nurture@kidneyresearchuk.org |
| Aachen | Peter Boor | Email: pboor@ukaachen.de |
| Leicester | Jonathan Barratt | Email: jb81@leicester.ac.uk |
| Rochester | Andrew Rule | Email: rule.andrew@mayo.edu |
| Diyarbakir | Ulaş Alabalik | Email: ulasalabalik@gmail.com |
| CureGN | Laura Barisoni for the CureGN investigators | Data are available upon request through the CureGN Ancillary Studies program. Data access is governed by the CureGN Steering Committee and NIDDK. Additional data sets will be provided to the NIDDK Central Repository after completion of study recruitment, which is currently ongoing. After data are deposited, the data will be available through the NIDDK Central Repository (https://repository.niddk.nih.gov/home/). |

**Supplementary Figure 1.** Baseline cumulative event probability (A) and overall treatment effect (B) in the pooled derivation and validation cohort (n = 1,022).


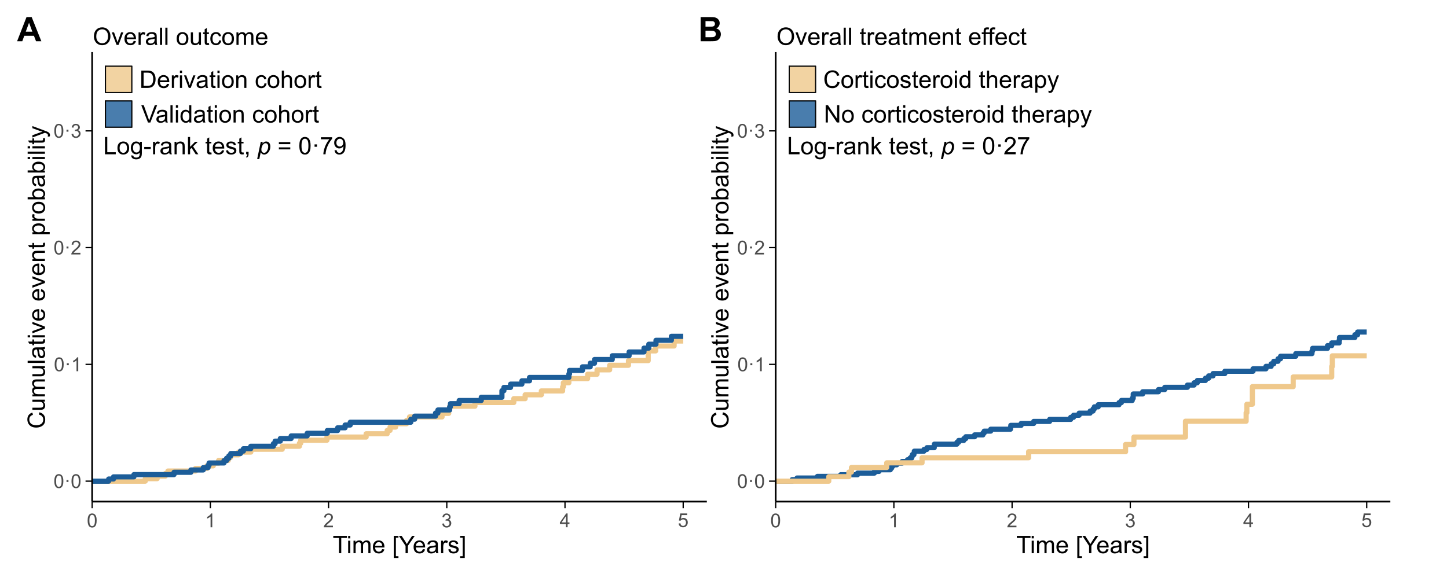


**Supplementary Figure 2.** Qini (A) and rank-weighted average treatment effect (B) for the Xboost model in the derivation cohort (n = 464). Patients were ranked based on their predicted individualised treatment benefit from most to least likely to benefit from corticosteroid treatment. The straight dotted line in (A) represents a random treatment allocation which is compared to the model’s predicted uplift. The computed Qini coefficient is the area between the solid curve and dotted line. Based on the increase in benefit for the grouped quantiles, an individualised treatment recommendation was derived in derivation and validation cohorts.


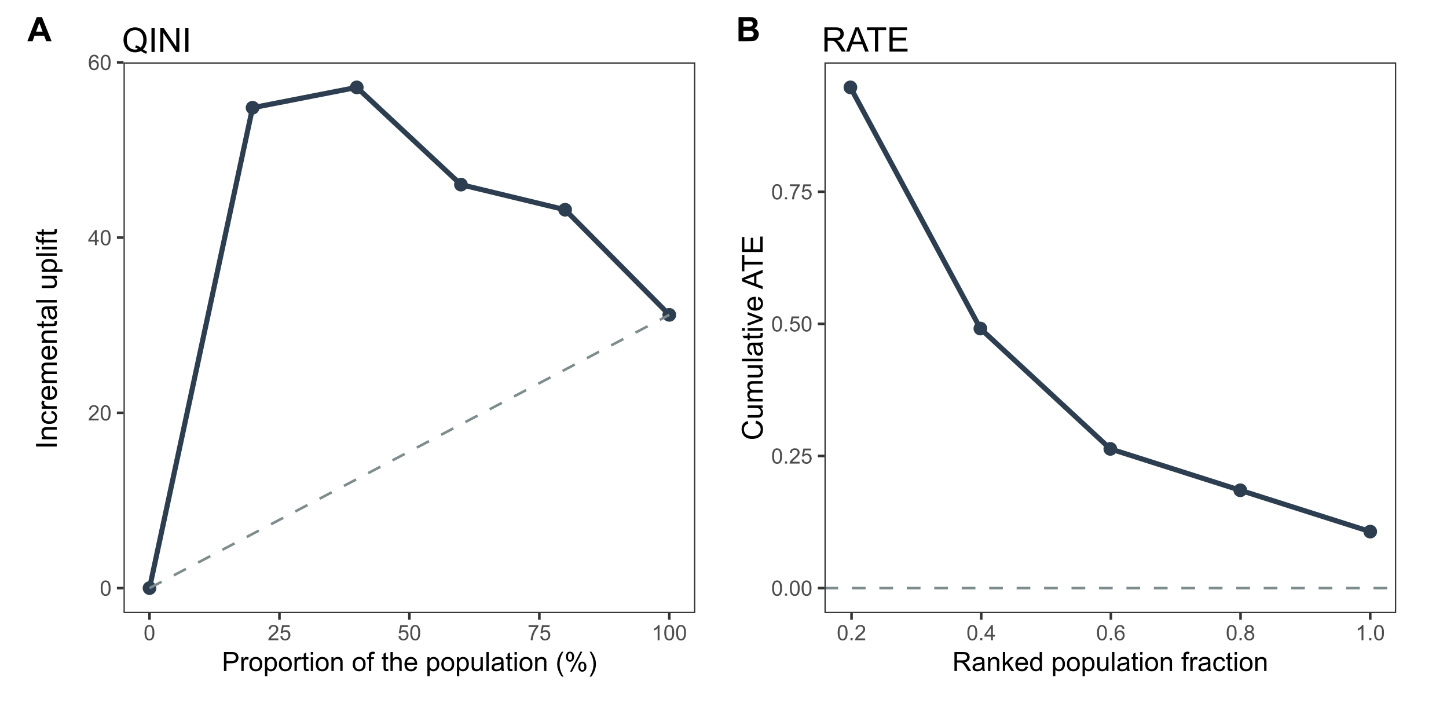


**Supplementary Figure 3.** Calibration of the Xboost model in the validation cohort (n = 558). Participants were stratified into tertiles based on predicted treatment effect. For each tertile, the x axis shows the mean predicted effect and the y axis shows the mean observed effect estimated with a normalised augmented inverse probability weighting (AIPW) estimator; vertical error bars indicate bootstrapped 95% confidence intervals for the estimated observed effect. Both axes were linearly normalised to for visualisation. The dashed diagonal denotes perfect calibration.


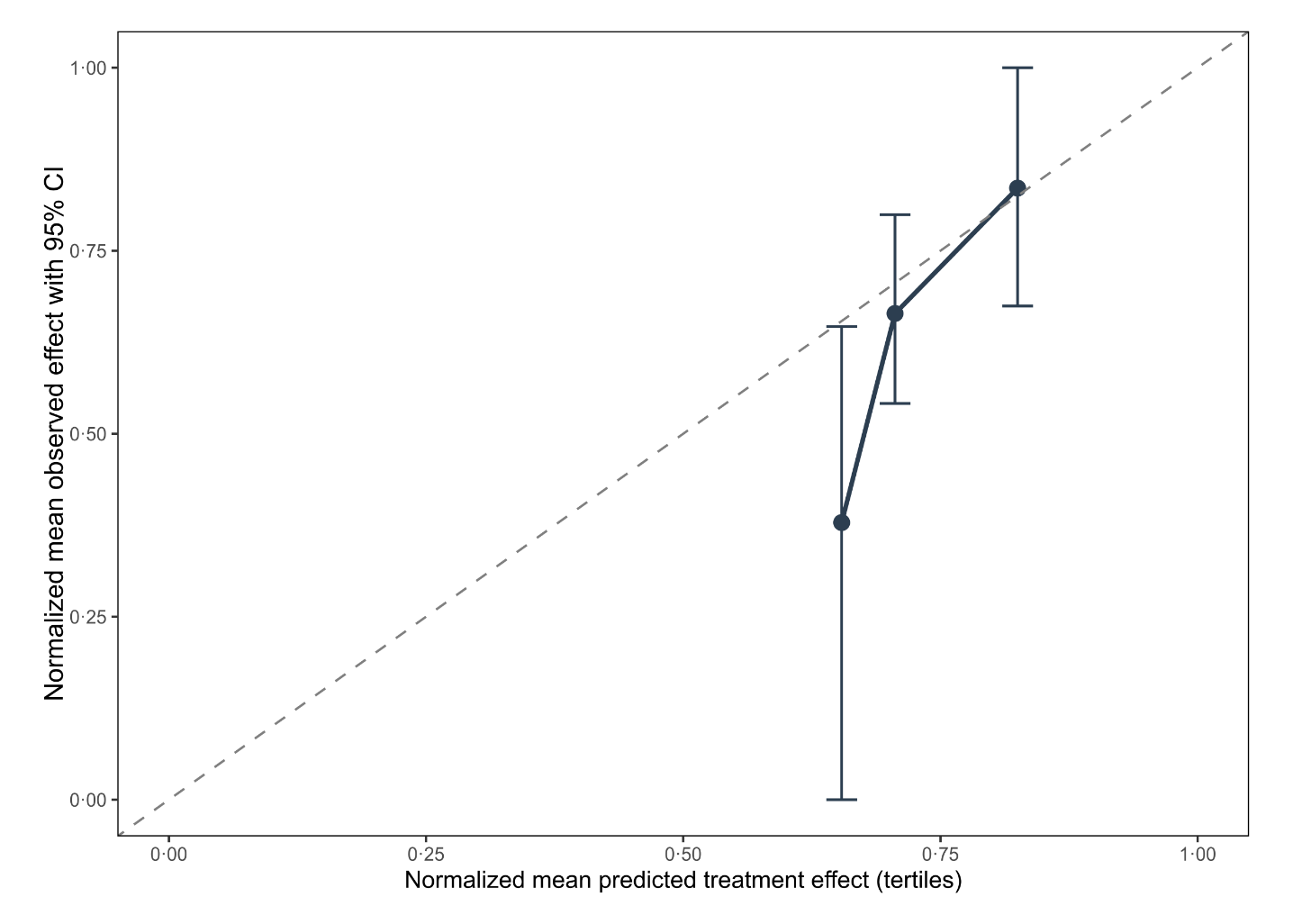


**Supplementary Figure 4.** Shapley additive explanations (SHAP) values of representative cases from the validation cohort with high (A-B) and low (C) predicted treatment benefit. The x-axis includes the six most important predictors which were sequentially added to the prediction to demonstrate the change from average treatment effect (ATE) to individualised treatment effect (ITE).

Abbreviations: gfr, estimated glomerular filtration rate; E, endocapillary hypercellularity; C, crescents.


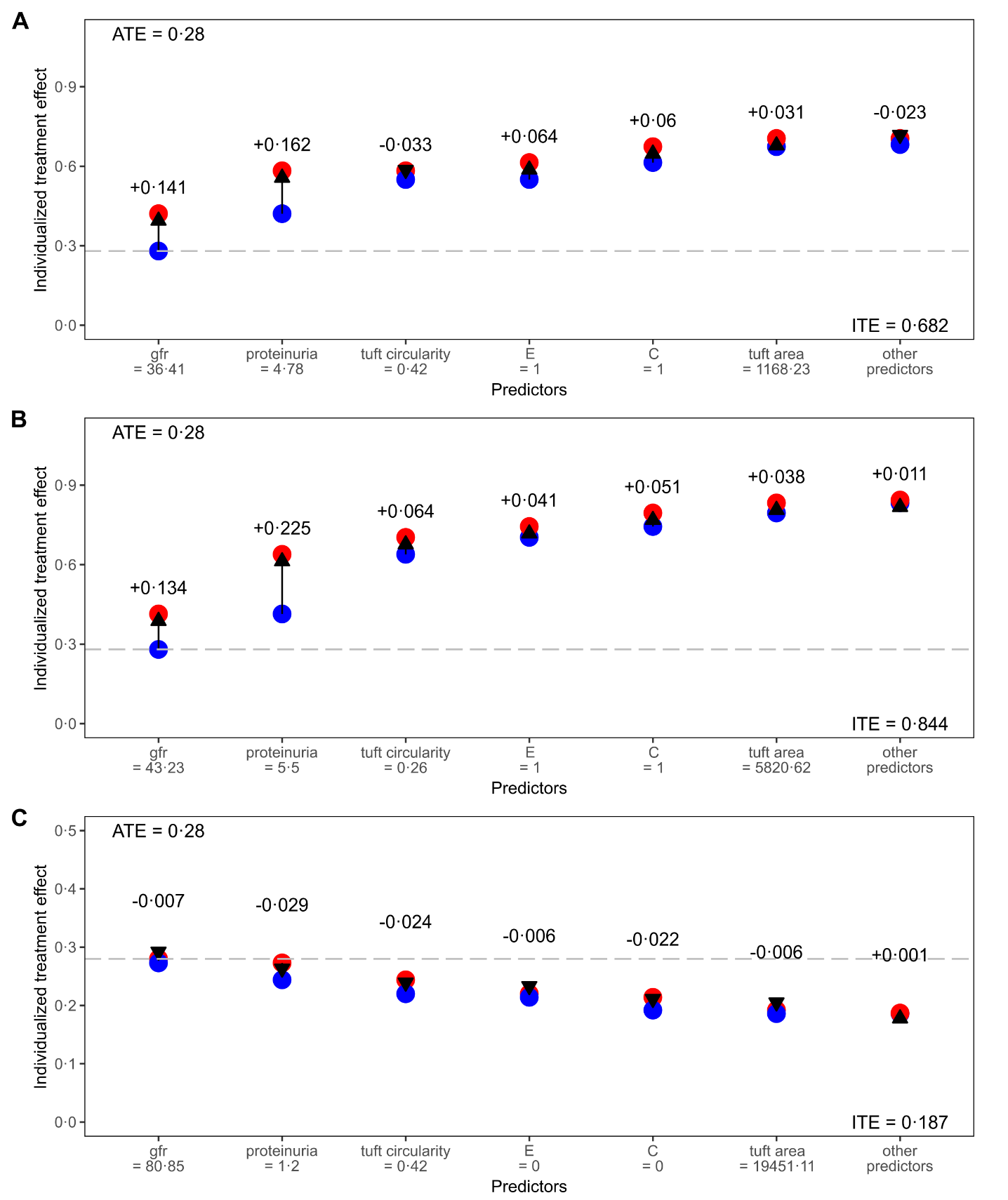


**Supplementary Figure 5.** Shapley additive explanations (SHAP) values of MEST-C predictors (A-E) calculated in the validation cohort (n = 558).

Abbreviations: M, mesangial hypercellularity; E, endocapillary hypercellularity; S, segmental glomerulosclerosis; T, tubular atrophy and interstitial fibrosis; C, crescents.


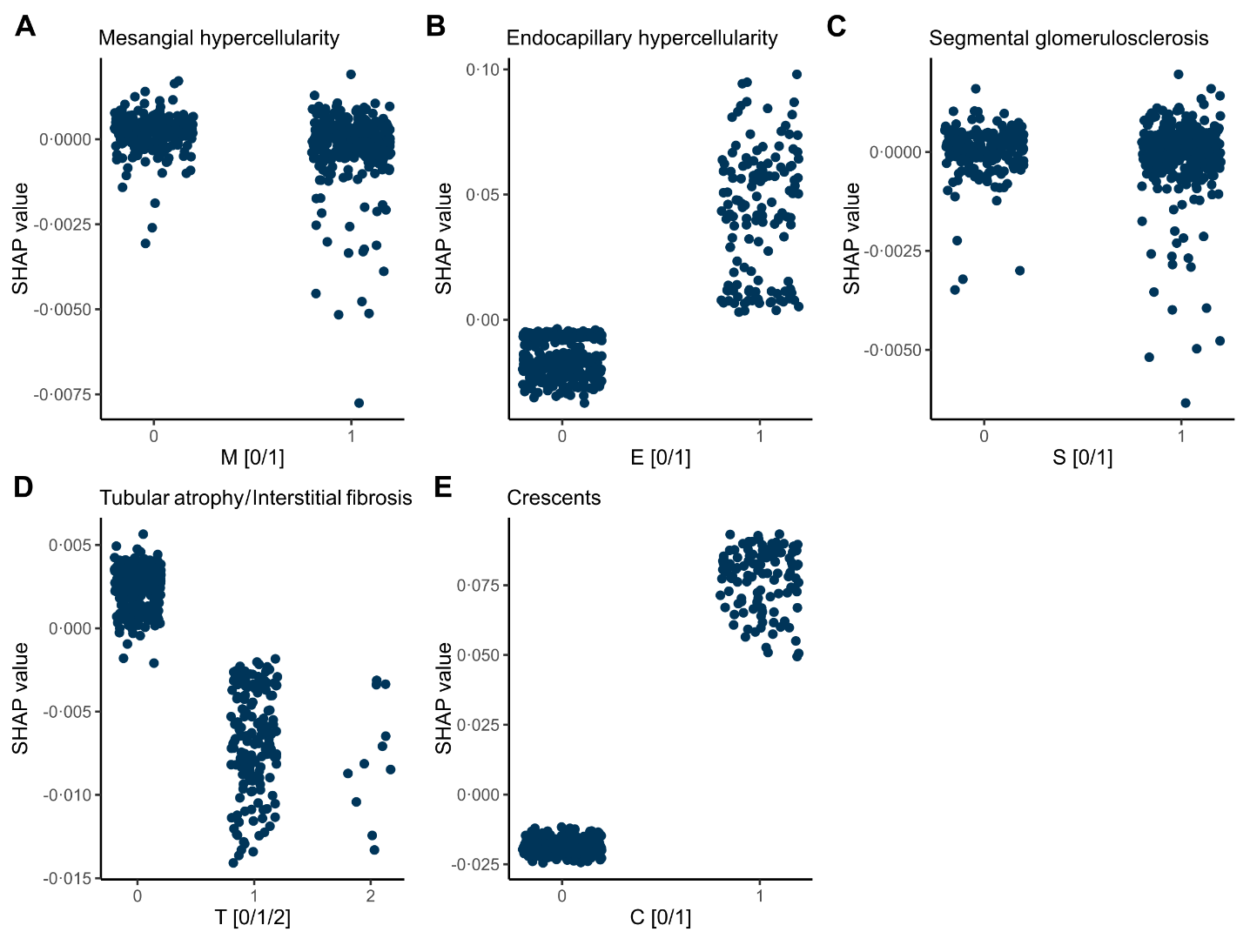


**Supplementary Figure 6.** Representative visualisations of glomeruli in patients with high (A) or low predicted treatment benefit (B) based on the associated glomerular tuft circularity. Scale bar: 100µm.


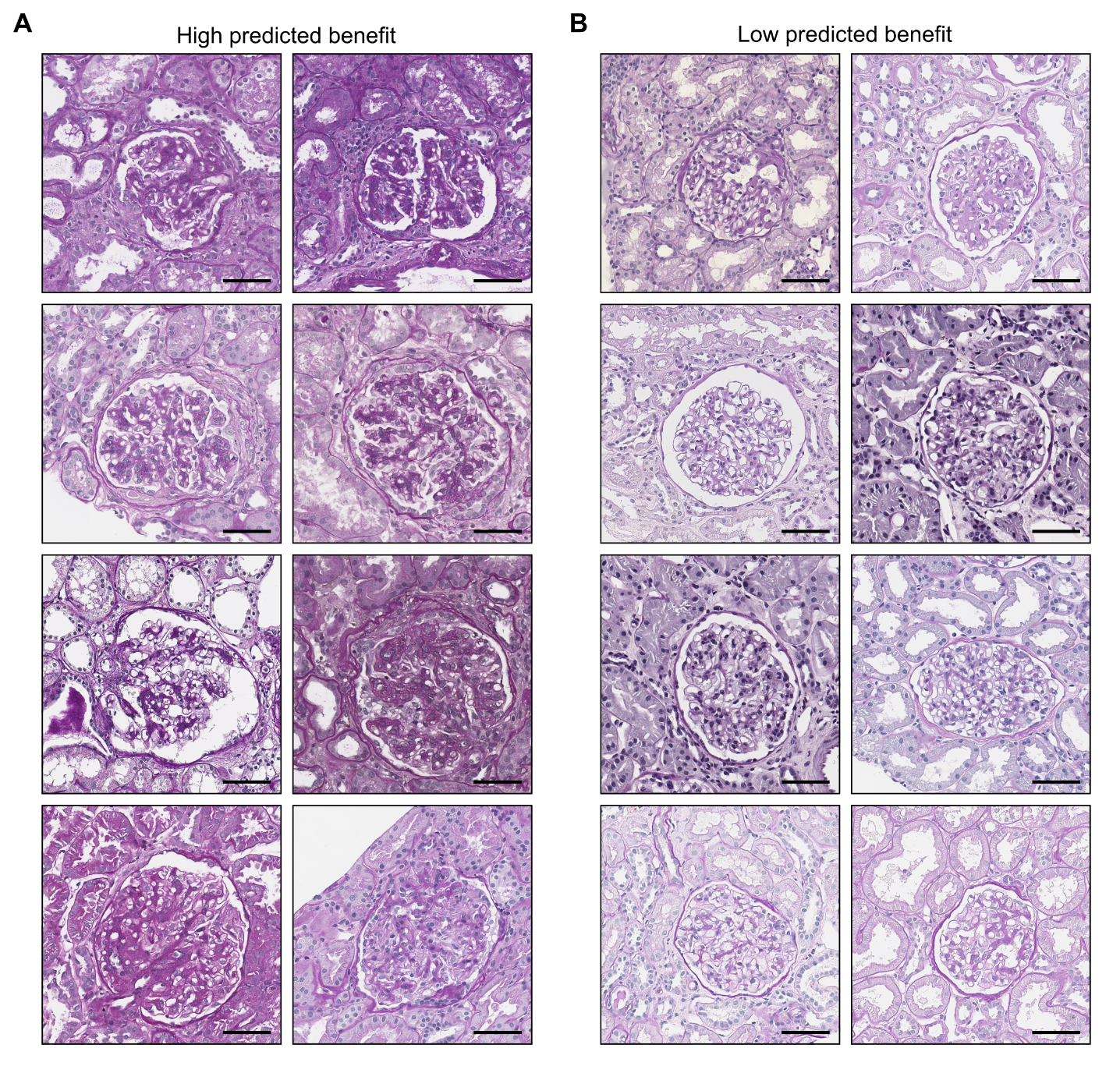


**Supplementary Figure 7.** Density plots of propensity score overlap by treatment groups in the validation cohort (n = 558). Visualisation of distributions for non-treated (blue, n = 446) and treated (yellow, n = 112) patients.

**
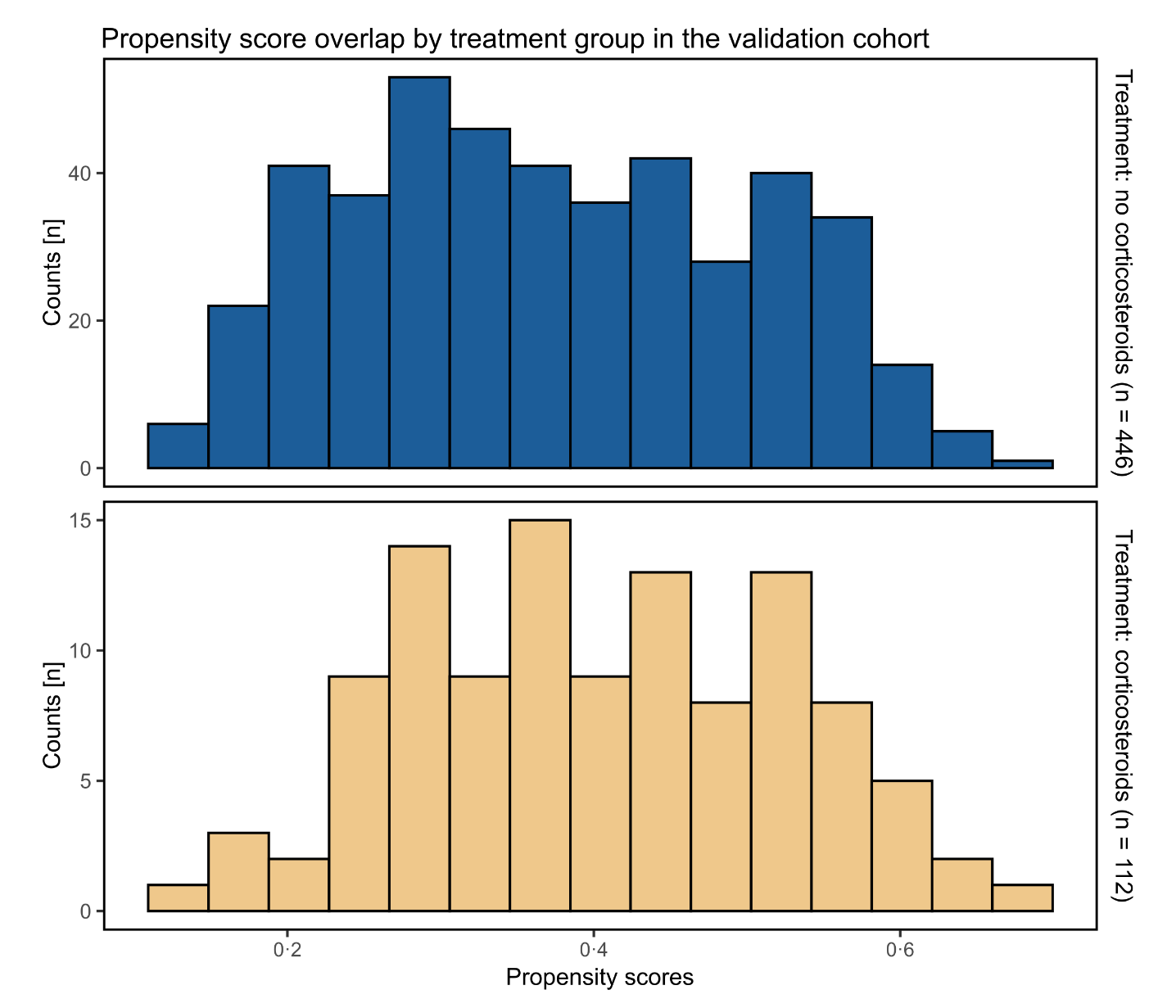
**

**Supplementary Figure 8.** Distribution of predicted individualised treatment effects in both derivation (n = 464) and validation cohort (n = 558; A), as well as in all subcohorts (VALIGA derivation n = 352, Kyoto n = 92, NURTuRE-CKD n = 20, VALIGA validation n = 155, Leicester n = 93, Diyarbakir n = 106, CureGN n = 135, Rochester n = 42, Aachen n = 27; B).


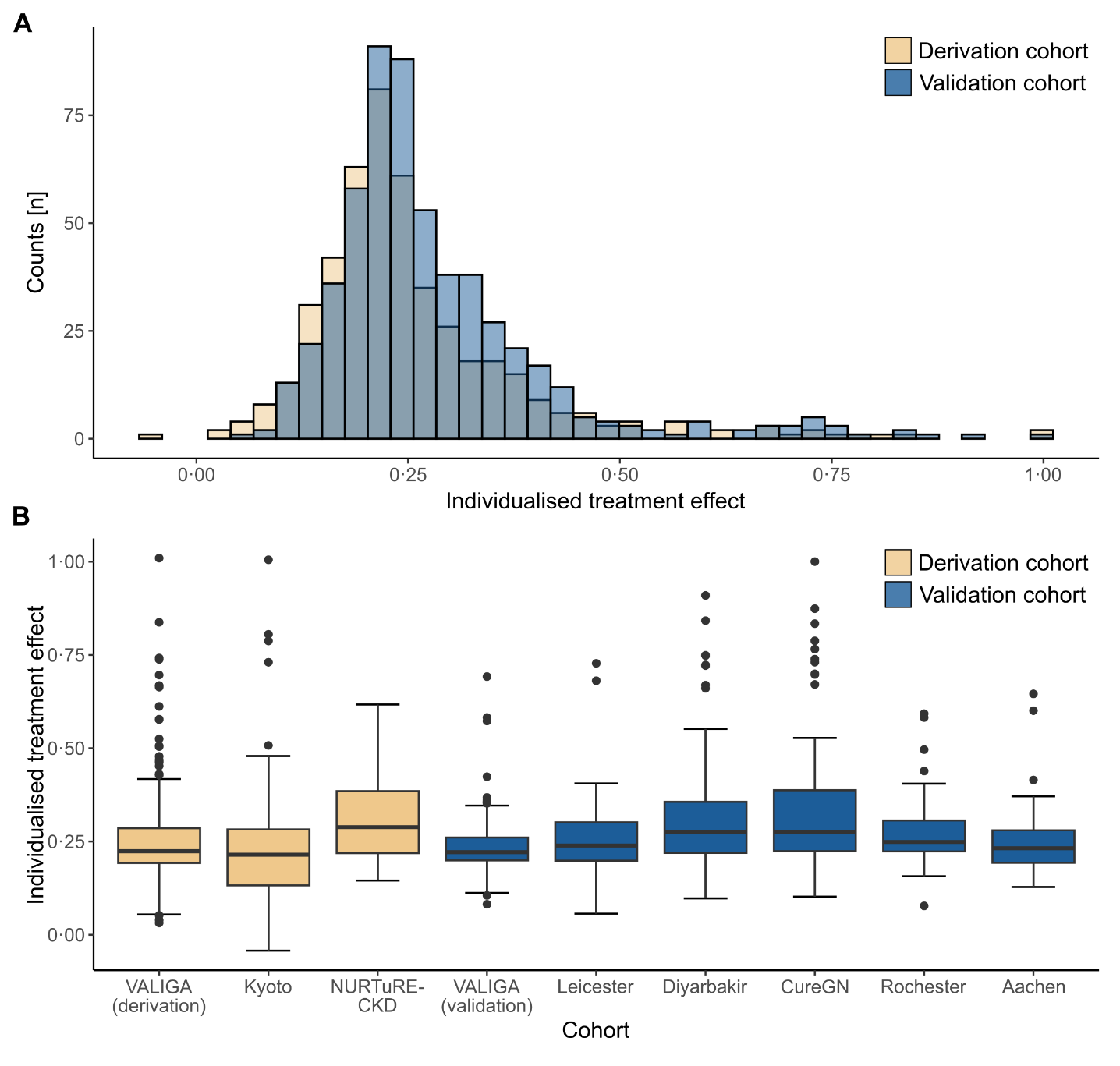

Supplement: Supplementary Tables and Figures [file mmc1.docx]
