# Supplementary material for: Individualised treatment effects of corticosteroids in IgA nephropathy
Source: eBioMedicine. 2026 Jul 14;130:106396. doi: 10.1016/j.ebiom.2026.106396 (PMC13377490; doi:10.1016/j.ebiom.2026.106396)
Supplement: VALIGA investigators [file mmc2.docx]

**VALIGA investigators**

| **First Name** | **Last Name** |
| --- | --- |
| M.L. | Russo |
| S. | Troyanov |
| H.T. | Cook |
| I.S.D | Roberts |
| V. | Tesar |
| D. | Maixnerova |
| S. | Lundberg |
| L. | Gesualdo |
| F. | Emma |
| F. | Diomedi |
| G. | Beltrame |
| C. | Rollino |
| A. | Amore |
| R. | Camilla |
| L. | Peruzzi |
| M. | Praga |
| S. | Feriozzi |
| R. | Polci |
| G. | Segoloni |
| L. | Colla |
| A. | Pani |
| D. | Piras |
| A. | Angioi |
| G. | Cancarini |
| S. | Ravera |
| M. | Durlik |
| E. | Moggia |
| J. | Ballarin |
| S. | Di Giulio |
| F. | Pugliese |
| I. | Serriello |
| Y. | Caliskan |
| M. | Sever |
| I. | Kilicaslan |
| F. | Locatelli |
| L. | Del Vecchio |
| J.F.M. | Wetzels |
| H. | Peters |
| U. | Berg |
| F. | Carvalho |
| A.C. | da Costa Ferreira |
| M. | Maggio |
| A. | Wiecek |
| M. | Ots-Rosenberg |
| R. | Magistroni |
| R. | Topaloglu |
| Y. | Bilginer |
| M. | D’Amico |
| M. | Stangou |
| F. | Giacchino |
| D. | Goumenos |
| P. | Kalliakmani |
| M. | Papasotiriou |
| K. | Galesic |
| C. | Geddes |
| K. | Siamopoulos |
| O. | Balafa |
| M. | Galliani |
| P. | Stratta |
| M. | Quaglia |
| R. | Bergia |
| R. | Cravero |
| M. | Salvadori |
| L. | Cirami |
| B. | Fellstrom |
| H. | Kloster Smerud |
| F. | Ferrario |
| T. | Stellato |
| J. | Egido |
| C. | Martin |
| J. | Floege |
| F. | Eitner |
| A. | Lupo |
| P. | Bernich |
| P. | Menè |
| M. | Morosetti |
| C. | van Kooten |
| T. | Rabelink |
| M.E.J. | Reinders |
| J.M. | Boria Grinyo |
| S. | Cusinato |
| L. | Benozzi |
| S. | Savoldi |
| C. | Licata |
| M. | Mizerska-Wasiak |
| G. | Martina |
| A. | Messuerotti |
| A. | Dal Canton |
| C. | Esposito |
| C. | Migotto |
| G. | Triolo |
| F. | Mariano |
| C. | Pozzi |
| R. | Boero |
| S. | Bellur |
| G. | Mazzucco |
| C. | Giannakakis |
| E. | Honsova |
| B. | Sundelin |
| A.M. | Di Palma |
| E. | Gutiérrez |
| A.M. | Asunis |
| J. | Barratt |
| R. | Tardanico |
| A. | Perkowska-Ptasinska |
| J. | Arce Terroba |
| M. | Fortunato |
| A. | Pantzaki |
| Y. | Ozluk |
| E. | Steenbergen |
| M. | Soderberg |
| Z. | Riispere |
| L. | Furci |
| D. | Orhan |
| D. | Kipgen |
| D. | Casartelli |
| D. | Galesic Ljubanovic |
| H. | Gakiopoulou |
| E. | Bertoni |
| P. | Cannata Ortiz |
| H. | Karkoszka |
| H.J. | Groene |
| A. | Stoppacciaro |
| I. | Bajema |
| J. | Bruijn |
| X. | Fulladosa Oliveras |
| J. | Maldyk |
| E. | Ioachim |
| N. | Bavbek |
| C. | Alpers |
| F. | Berthoux |
| S. | Bonsib |
| V. | D’Agati |
| G. | D’Amico |
| S. | Emancipator |
| F. | Emmal |
| F. | Fervenza |
| S. | Florquin |
| A. | Fogo |
| M. | Haas |
| P. | Hill |
| R. | Hogg |
| S. | Hsu |
| T. | Hunley |
| M. | Hladunewich |
| C. | Jennette |
| K. | Joh |
| B. | Julian |
| T. | Kawamura |
| F. | Lai |
| C. | Leung |
| L. | Li |
| P. | Li |
| Z. | Liu |
| A. | Massat |
| B. | Mackinnon |
| S. | Mezzano |
| F. | Schena |
| Y. | Tomino |
| P. | Walker |
| H. | Wang |
| J. | Weening |
| N. | Yoshikawa |
| C.H. | Zeng |
| S. | Shi |
| C. | Nogi |
| H. | Suzuki |
| K. | Koike |
| K. | Hirano |
| T. | Kawamura |
| T. | Yokoo |
| M. | Hanai |
| K. | Fukami |
| K. | Takahashi |
| Y. | Yuzawa |
| M. | Niwa |
| Y. | Yasuda |
| S. | Maruyama |
| D. | Ichikawa |
| T. | Suzuki |
| S. | Shirai |
| A. | Fukuda |
| S. | Fujimoto |
| H. | Trimarchi |
