# Supplementary material for: Individualised treatment effects of corticosteroids in IgA nephropathy
Source: eBioMedicine. 2026 Jul 14;130:106396. doi: 10.1016/j.ebiom.2026.106396 (PMC13377490; doi:10.1016/j.ebiom.2026.106396)
Supplement: NURTuRE investigators [file mmc3.docx]

**The National Unified Renal Translational Research Enterprise (NURTuRE) academic steering group members**

| **First name** | **Last name** |
| --- | --- |
| Maarten W. | Taal |
| Paul | Cockwell |
| Simon D.S. | Fraser |
| Philip A. | Kalra |
| Moin | Saleem |
| David C. | Wheeler |
