# Supplementary material for: Individualised treatment effects of corticosteroids in IgA nephropathy
Source: eBioMedicine. 2026 Jul 14;130:106396. doi: 10.1016/j.ebiom.2026.106396 (PMC13377490; doi:10.1016/j.ebiom.2026.106396)
Supplement: AI4IgAN investigators [file mmc4.docx]

**AI4IgAN investigators**

| **First name** | **Last name** |
| --- | --- |
| Rosanna | Coppo |
| Sean | Barbour |
| Jonathan | Barratt |
| Ian S.D. | Roberts |
| Juergen | Floege |
| Vladimir | Tesar |
| Roman D. | Bülow |
| David L. | Hölscher |
| Hong | Zhang |
| Muh Geot | Wong |
| Laura | Barisoni |
| Mark | Haas |
| Motoko | Yanagita |
| Keiichi | Kaneko |
| Takeo | Koshida |
| Sigrid | Lundberg |
