# Supplementary material for: Individualised treatment effects of corticosteroids in IgA nephropathy
Source: eBioMedicine. 2026 Jul 14;130:106396. doi: 10.1016/j.ebiom.2026.106396 (PMC13377490; doi:10.1016/j.ebiom.2026.106396)
Supplement: CureGN investigators [file mmc5.docx]

**CureGN investigators**

| **First Name** | **Last Name** |
| --- | --- |
| Gerald | Appel |
| Revekka | Babayev |
| Ibrahim | Batal |
| Andrew | Bomback |
| Pietro | Canetta |
| Brenda | Chan |
| Vivette Denise | D'Agati |
| Samitri | Dogra |
| Hilda | Fernandez |
| Gabriele | Gaggero |
| Ali | Gharavi |
| William | Hines |
| Krzysztof | Kiryluk |
| Satoru | Kudose |
| Fangming | Lin |
| Victoria | Kolupaeva |
| Maddalena | Marasa |
| Glen | Markowitz |
| Mariela | Navarro-Torres |
| Hila Milo | Rasouly |
| Sumit | Mohan |
| Nicola | Mongera |
| Jordan | Nestor |
| Jai | Radhakrishnan |
| Maya | Rao |
| Maya | Sabatello |
| Simone | Sanna-Cherchi |
| Dominick | Santoriello |
| Miroslav | Sekulic |
| Michael Barry | Stokes |
| Natalie | Uy |
| Natalie | Vena |
| Benjamin | Wooden |
| Bartosz | Foroncewicz |
| Natalia | Wiewiórska-Krata |
| Barbara | Moszczuk |
| Krzysztof | Mucha |
| Agnieszka | Perkowska-Ptasińska |
| Elżbieta | Ryszkowska |
| Francesca | Lugani |
| Valerio | Vellone |
| Diego | Aviles |
| Tarak | Srivastava |
| Alexander | Katz |
| Sun-Young | Ahn |
| Prasad | Devarajan |
| Elif | Erkan |
| Hillarey | Stone |
| Sherene | Mason |
| Liliana | Gomez-Mendez |
| Larry | Greenbaum |
| Chia-shi | Wang |
| Hong (Julie) | Yin |
| Jens | Goebel |
| Donald | Weaver |
| Jill | Krissberg |
| Jerome | Lane |
| Cindy | Pan |
| Ellen | Cody |
| Samantha | Martinek-Bundt |
| Dawson | Carmean |
| Mary | Dreher |
| Mahmoud | Kallash |
| John | Mahan |
| Samantha | Sharpe |
| William | Smoyer |
| Laura | Biederman |
| Amira | Al-Uzri |
| Sandra | Iragorri |
| Myda | Khalid |
| Craig | Belsha |
| Elizabeth | Onugha |
| Michael | Braun |
| A.C. | Gomez |
| Tetyana | Vasylyeva |
| Daniel | Feig |
| Melisha | Hannah |
| Aftab | Chishti |
| Jon | Klein |
| Chryso | Katsoufis |
| Wacharee | Seeherunvong |
| Michelle | Rheault |
| Craig | Wong |
| Qassim | Abid |
| John | Barcia |
| Agnes | Swiatecka-Urban |
| Sharon | Bartosh |
| Brian | Stotter |
| Joseph | Gaut |
| Louis-Philippe | Laurin |
| Virginie | Royal |
| Mathieu | Latour |
| Natlie (Natacha) | Patey |
| Anand | Achanti |
| Milos | Budisavljevic |
| Vishwajeeth | Pasham |
| Cybele | Ghossein |
| Yonatan | Peleg |
| Salem | Almaani |
| Isabelle | Ayoub |
| Samir | Parikh |
| Brad | Rovin |
| Anjali | Satoskar |
| Anthony | Chang |
| Huma | Fatima |
| Jan | Novak |
| Matthew | Renfrow |
| Dana | Rizk |
| Dhruti | Chen |
| Vimal | Derebail |
| Ronald | Falk |
| Keisha | Gibson |
| Dorey | Glenn |
| Susan | Hogan |
| Koyal | Jain |
| J. Charles | Jennette |
| Vanessa | Moreno |
| Amy | Mottl |
| Caroline | Poulton |
| Monica | Reynolds |
| Manish Kanti | Saha |
| Nicole E. | Wyatt |
| Agnes | Fogo |
| Neil | Sanghani |
| Jason | Kidd |
| Selvaraj | Muthusamy |
| Rebecca | Scobell |
| Michelle | Denburg |
| Amy | Kogon |
| Kevin | Meyers |
| Madhura | Pradhan |
| Raed | Bou Matar |
| John | O'Toole |
| John | Sedor |
| Christine | Sethna |
| Suzanne | Vento |
| Mohamed | Atta |
| Serena | Bagnasco |
| Alicia | Neu |
| John | Sperati |
| Sharon | Adler |
| Tiane | Dai |
| Ram | Dukkipati |
| Frederick | Kaskel |
| Kaye | Brathwaite |
| Kimberly | Reidy |
| Laura | Malaga-Dieguez |
| Katherine | Tuttle |
| Richard | Lafayette |
| Kamal | Fahmeedah |
| Elizabeth | Talley |
| Michelle | Hladunewich |
| Rulan | Parekh |
| Carmen | Avila-Casado |
| Daniel | Cattran |
| Heather | Reich |
| Meherzad | Kutky |
| Yelena | Drexler |
| Alessia | Fornoni |
| Jeffrey | Hodgin |
| Andrea | Oliverio |
| Jon | Hogan |
| Lawrence | Holzman |
| Matthew | Palmer |
| Gaia | Coppock |
| Michael | Mortiz |
| Juhi | Kumar |
| Charles | Alpers |
| J. Ashley | Jefferson |
| Kamal | Sambandam |
| Bethany | Roehm |
| Cynthia | Nast |
| Jean | Hou |
| Laura | Barisoni |
| Crystal | Gadegbeku |
| Abigail | Smith |
| Brenda | Gillespie |
| Bruce | Robinson |
| Matthias | Kretzler |
| Zubin | Modi |
| Laura | Mariani |
| Lisa M. | Guay-Woodford |
